# Nitric Oxide-Scavenging, Anti-Migration Effects, and Glycosylation Changes after Hemin Treatment of Human Triple-Negative Breast Cancer Cells: A Mechanistic Study

**DOI:** 10.1021/acsptsci.3c00115

**Published:** 2023-09-11

**Authors:** Amir M. Alsharabasy, Amal Aljaabary, Raghvendra Bohara, Pau Farràs, Sharon A. Glynn, Abhay Pandit

**Affiliations:** † CÚRAM, SFI Research Centre for Medical Devices, University of Galway, Galway H91 W2TY, Ireland; ‡ School of Biological and Chemical Sciences, Ryan Institute, University of Galway, Galway H91 TK33, Ireland; § Discipline of Pathology, Lambe Institute for Translational Research, School of Medicine, University of Galway, Galway H91 YR71, Ireland

**Keywords:** triple-negative breast cancer, nitric oxide, hemin, nitrosylation, metastasis, glycoprotein

## Abstract

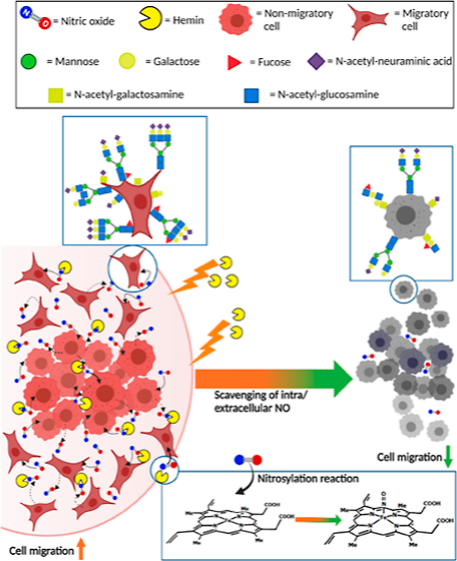

The enhanced expression of nitric oxide (^•^NO) synthase predicts triple-negative breast cancer outcome and its resistance to different therapeutics. Our earlier work demonstrated the efficiency of hemin to scavenge the intra- and extracellular ^•^NO, proposing its potency as a therapeutic agent for inhibiting cancer cell migration. In continuation, the present work evaluates the effects of ^•^NO on the migration of MDA-MB-231 cells and how hemin modulates the accompanied cellular behavior, focusing on the corresponding expression of cellular glycoproteins, migration-associated markers, and mitochondrial functions. We demonstrated for the first time that while ^•^NO induced cell migration, hemin contradicted that by ^•^NO-scavenging. This was in combination with modulation of the ^•^NO-enhanced glycosylation patterns of cellular proteins with inhibition of the expression of specific proteins involved in the epithelial–mesenchymal transition. These effects were in conjunction with changes in the mitochondrial functions related to both ^•^NO, hemin, and its nitrosylated product. Together, these results suggest that hemin can be employed as a potential anti-migrating agent targeting ^•^NO-scavenging and regulating the expression of migration-associated proteins.

Triple-negative breast cancer (TNBC) is one of the most aggressive subtypes of breast cancer^1^ and is characterized by early recurrence within 2–3 years of first diagnosis.^2^ Additionally, heterogeneity of the TNBC tissue creates a difficulty in developing the treatments for the different TNBC subtypes.^3^ The development of highly invasive cancer cell phenotypes has been reported to be correlated with alterations in the expression and branching of various cell surface glycoproteins.^4,5^ For instance, the production of shorter and more branched fucosylated, sialylated, and sulfated glycans was reported in different malignant cells.^6−8^ These altered glycans enhance tumor cell growth, adhesion, and migration.^9,10^ Moreover, the targeting of certain metastasis-promoting glycotopes enhanced the effects of both radio-and chemotherapy in certain cancers.^11−13^

In patients with TNBC, there is a correlation between the tumor progression and overexpression of inducible nitric oxide synthase (iNOS).^14−16^ For instance, the iNOS selective inhibitor, aminoguanidine, inhibited the metastasis of MDA-MB-231 from the fat pad to the brain in a mouse model for TNBC, with a decrease in cell resistance to Paclitaxel.^17^ However, although different iNOS selective inhibitors promoted the potential of chemotherapeutic agents in combination with other drugs, some did not work with radiotherapy.^18,19^ Moreover, the increased levels of ^•^NO within the tumor microenvironment arise from both the tumor and host tissues. Hence, applying the NOS-inhibition strategy can be adjusted and tuned according to the tumor tissue type and the major ^•^NO-sources. Therefore, targeting ^•^NO within the tumor cells can be an alternative approach to overcome some of the problems of NOS inhibition.

In our recent article, we reported the affinity of hemin towards binding ^•^NO and its oxidation into nitrite, and how this implicates the nitration of intracellular proteins in MDA-MB-231 cells.^20^ Moreover, we demonstrated the effects of hemin and a number of its derivatives on the migration of the TNBC cells: MDA-MB-231 and HCC1806 cells.^21^ In addition, these effects were comparable to those of aminoguanidine.^21^ Although these results refer to the promising application of hemin and/or one of its derivatives as anti-cancer agents, a mechanistic study of how hemin can modulate cell migration is required.

The following study started with evaluating hemin’s effects on the ^•^NO-levels in solution by ultraviolet–visible (UV–vis) spectroscopy. Next, we performed a cell-based study to evaluate the effects of ^•^NO-scavenging by hemin on MDA-MB-231 cells and compare to those elicited by different concentrations of ^•^NO and the effects of hemin only via the following approaches: (1) evaluation of MDA-MB-231 cell migration; (2) studying of the changes in expression of some cell surface glycoproteins by lectin staining; (3) measurement of the accompanied change in expression of cluster of differentiation 44 (CD44), heme-oxygenase (HOX-1), matrix metallopeptidase 14 (MMP-14), hypoxia-inducible factor 1-alpha (HIF-α), and the epithelial–mesenchymal transition markers vimentin, vascular endothelial–cadherin (VE-cadherin), and epithelial cadherin (E-cadherin), and (4) investigation of the associated changes in mitochondrial functions.

## Results

### Decomposition of the ^•^NO-Donor Depends on the Testing Solution and ^•^NO Nitrosylates Hemin

Figure S1 shows an example of the changes in voltage due to 30 and 300 μM diethylenetriamine NONOate (DETA-NO) in both media. Different kinetics of ^•^NO-release from 30, 100, 300, 600, and 1000 μM DETA-NO in fetal bovine serum (FBS)-containing medium (Figure S2A) and phosphate buffer (Figure S2B) were observed. The accompanied temporal changes in the voltage signal are illustrated in (Figure S3A,B). In the presence of ^•^NO, the hemin absorption at 382 nm (A_382_) decreased, and this depended on the DETA-NO concentration and the incubation period (Figure S2C,D). The changes in the UV–vis spectra following hemin titration against 30, 100, 300 and 1000 μM DETA-NO are shown in Figure S4A–D.

### ^•^NO Enhances the Migration of MDA-MB-231 Cells, Which is Inhibited in the Presence of Hemin

**Figure 1:**
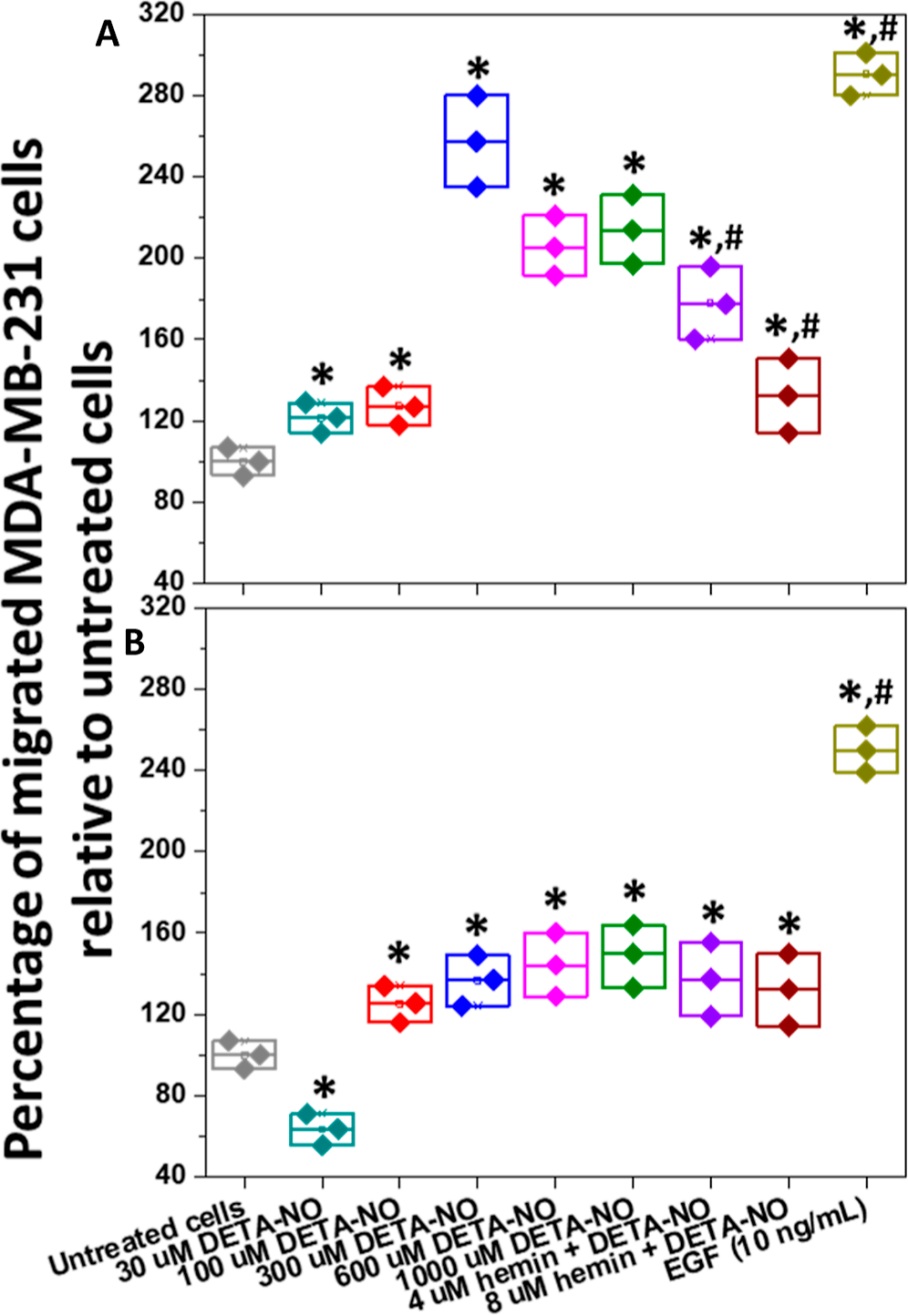
Without affecting cell invasion, hemin inhibits the ^•^NO-induced MDA-MB-231 cell migration through transwell membranes. The effects of EGF, different concentrations of DETA-NO and/or hemin on cell migration (A), and invasion through the layer (B) were evaluated. Results are expressed as the percentage of migrated cells normalized to the count in the control group (untreated cells migrated toward the medium only). EGF was employed as the positive control. Results are presented as mean ± S.D, *n* = 3. *P < 0.05 versus the untreated cells (negative control); #*P* < 0.05 versus the cells treated with 300 μM DETA-NO only using a two-tailed unpaired student *t*-test. “See also Figure S5”.

After 24 h of treatment, both epidermal growth factor (EGF) and ^•^NO, released from different concentrations of DETA-NO, enhanced the MDA-MB-231 cell migration significantly, and the maximum rate was at 300 μM DETA-NO, which decreased at higher concentrations (Figures 1A and S5A). Moreover, these concentrations significantly enhanced the cell invasion through collagen coating, starting by the 100 μM DETA-NO upward (Figures 1B and S5C). However, DETA-NO at 30 μM showed inhibition of cell invasion. In addition, while hemin contradicted the effects of ^•^NO and inhibited cell migration (Figures 1A and S5B), it did not cause any substantial changes to the ^•^NO-induced invasion (Figures 1B and S5D).

### Effects of ^•^NO and Hemin on Cell Migration are Accompanied by Alterations in Glycan Profiles

**Figure 2:**
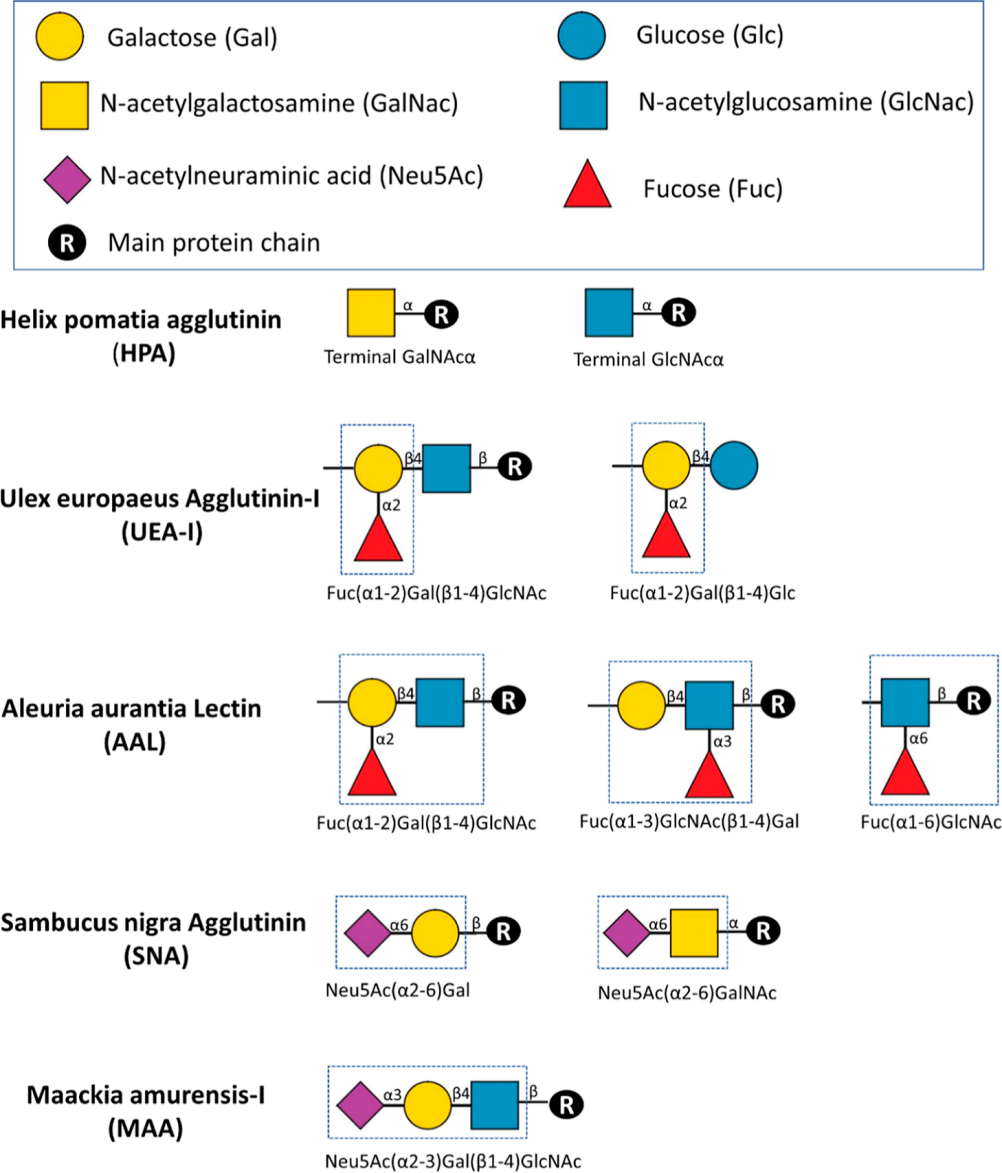
Different lectins employed in lectin staining and blotting and the N-glycans recognized by them. The determinants required for binding are indicated in the dotted blue boxes. The top box shows the symbolic representation of the monosaccharides illustrated.

Figure 2 summarizes the binding motifs of the used lectins.^22,23^ Via comparing of the fluorescence intensity of lectin-binding surface proteins after cell treatment with freshly prepared or degraded 300 μM DETA-NO (Table S1), only active DETA/NO impacted the lectin-binding (Figure S6A,B).

**Figure 3:**
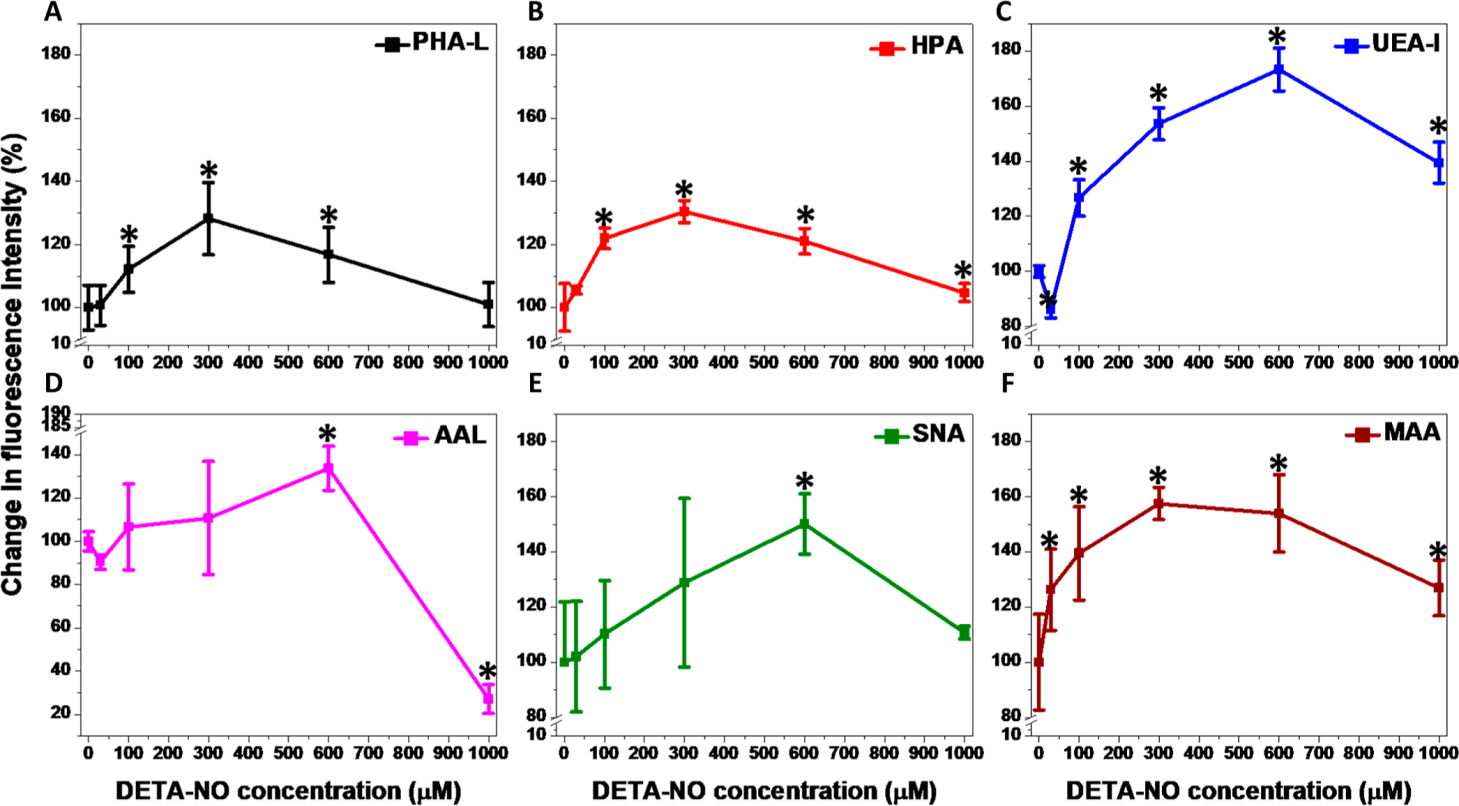
Effects of increasing concentrations of ^•^NO released from DETA-NO on the reactivity of MDA-MB-231 cell surface glycans. The different glycans were detected with the lectins: PHA-L (A), HPA (B), UEA-I (C), AAL (D), SNA (E), and MAA (F). Data are expressed as the percentage of change in the mean fluorescence intensity for each lectin under each treatment relative to the values obtained in untreated cells, set at 100%. Results are presented as mean ± S.D, *n* = 3. *P < 0.05 versus the untreated cells using a two-tailed unpaired student *t*-test. “See also Figures S7–S12”.

The released ^•^NO increased the levels of glycosylated cellular proteins, particularly the formation of branched tri and tetra-antennary complex N-linked glycans (Figures 3A and S7) and N-acetylgalactosamine glycans (Figures 3B and S8). These effects were proportional to the ^•^NO concentration. Moreover, the fluorescence intensity corresponding to *Ulex europaeus* Agglutinin I (UEA) (Figures 3C and S9) and *Aleuria aurantia* (AAL)-binding glycotopes (Figures 3D and S10) significantly decreased only in 30 μM DETA-NO-treated cells, followed by a gradual enhancement at higher concentrations. In addition, the cell treatment with different DETA-NO concentrations increased the fluorescence intensity of both *Sambucus nigra* Lectin (SNA) (Figures 3E and S11) and Maackia Amurensis Lectin I (MAA)-binding glycotopes (Figures 3F and S12). These changes were significant in case of the MAA-binding proteins in response to different DETA-NO concentrations, while only 600 μM DETA-NO significantly improved the SNA-binding glycotope formation.

**Figure 4:**
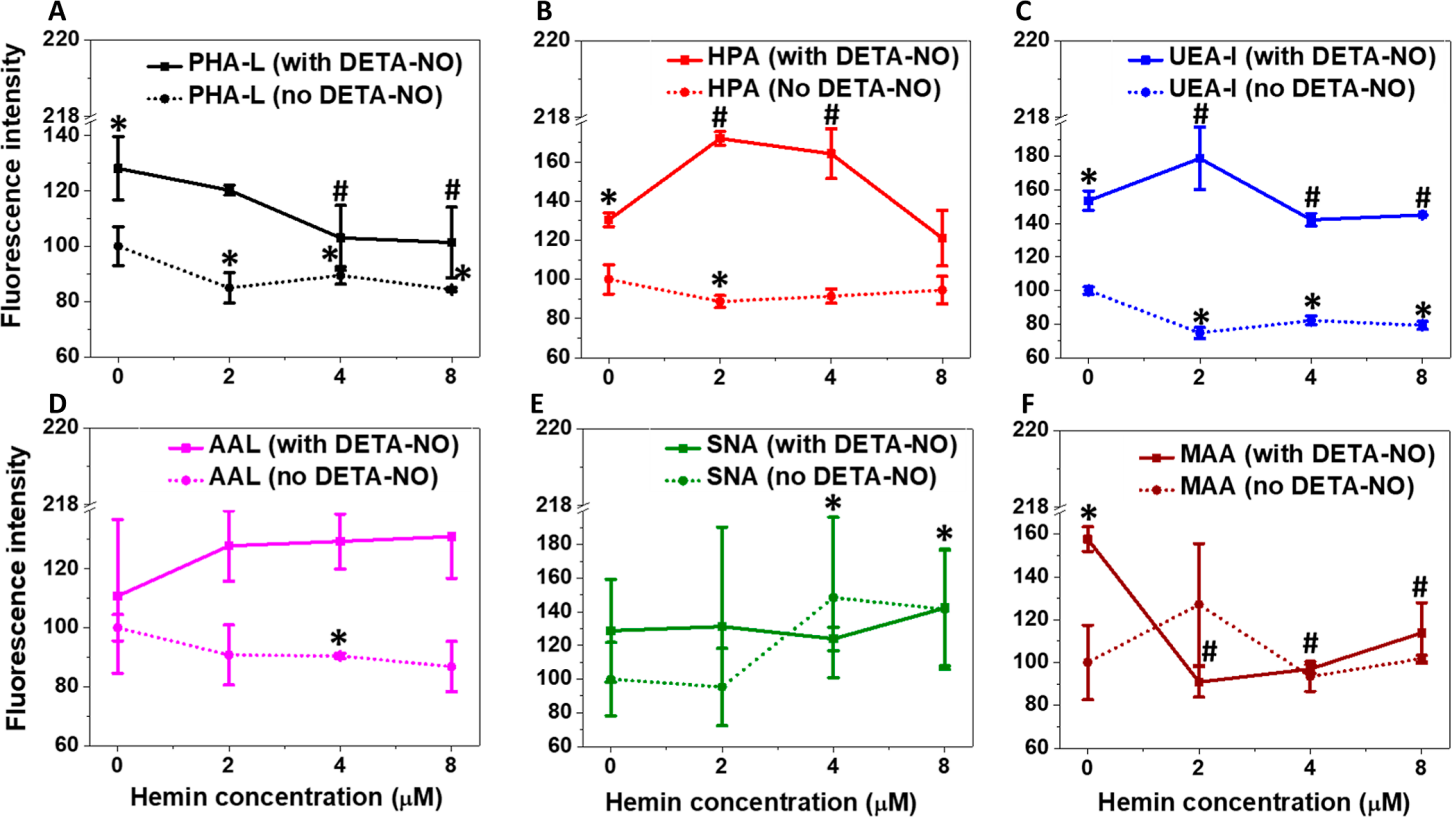
Effects of hemin in the presence and absence of ^•^NO from 300 μM DETA-NO on the reactivity of MDA-MB-231 cell surface glycans. The different glycans were detected with the lectins: PHA-L (A), HPA (B), UEA-I (C), AAL (D), SNA (E), and MAA (F). Data are expressed as the percentage of change in the mean fluorescence intensity for each lectin under each treatment relative to the values obtained in untreated cells, set at 100%. Results are presented as mean ± S.D, *n* = 3. *^,^#*P* < 0.05 versus the untreated and DETA-NO only treated cells (no added hemin) using a two-tailed unpaired student *t*-test. “See also Figures S13–S18”.

However, the cell treatment with hemin significantly reduced the fluorescence of *Phaseolus vulgaris* Leucoagglutinin (PHA-L)-binding glycotopes, in the presence and absence of ^•^NO (Figures 4A and S13). Nevertheless, while similar effects were observed in the *Helix pomatia* agglutinin (HPA)-binding glycotopes in ^•^NO-free cultures, hemin nitrosylation at 2 and 4 μM increased the fluorescence intensity (Figures 4B and S14). A slight, but significant decrease in the fluorescence corresponding UEA-I (Figures 4C and S15) and AAL-binding proteins (Figures 4D and S16) was detected in hemin only-treated cells. However, in the presence of ^•^NO, the fluorescence increased under both cases, but with different levels. UEA-binding protein expression was significantly enhanced in DETA-NO/2 μM hemin-treated cells, with no differences in the case of other hemin concentrations. Although similar results were detected with the AAL-binding proteins, the increase in fluorescence in DETA-NO/2 μM and DETA-NO/4 μM-treated cells was not significant, and only 8 μM hemin inhibited the effects of ^•^NO and reduced the fluorescence intensity sharply. Furthermore, in the absence of ^•^NO, cell treatment with hemin slightly changed the fluorescence intensity of both SNA (Figures 4E and S17) and MAA-binding glycotopes (Figures 4F and S18), with similar effects on the former glycotopes in the presence of ^•^NO. However, when cells were treated with DETA-NO and hemin, this decreased the fluorescence of MAA-binding glycotopes significantly with similar effects of 2 and 4 μM hemin, which were lower than that observed at DETA-NO/8 μM hemin.

### ^•^NO, Hemin, and the Product of Their Reaction Regulate the Expression of Different Proteins Involved in Cell Migration Dependent on Its Flux

**Figure 5:**
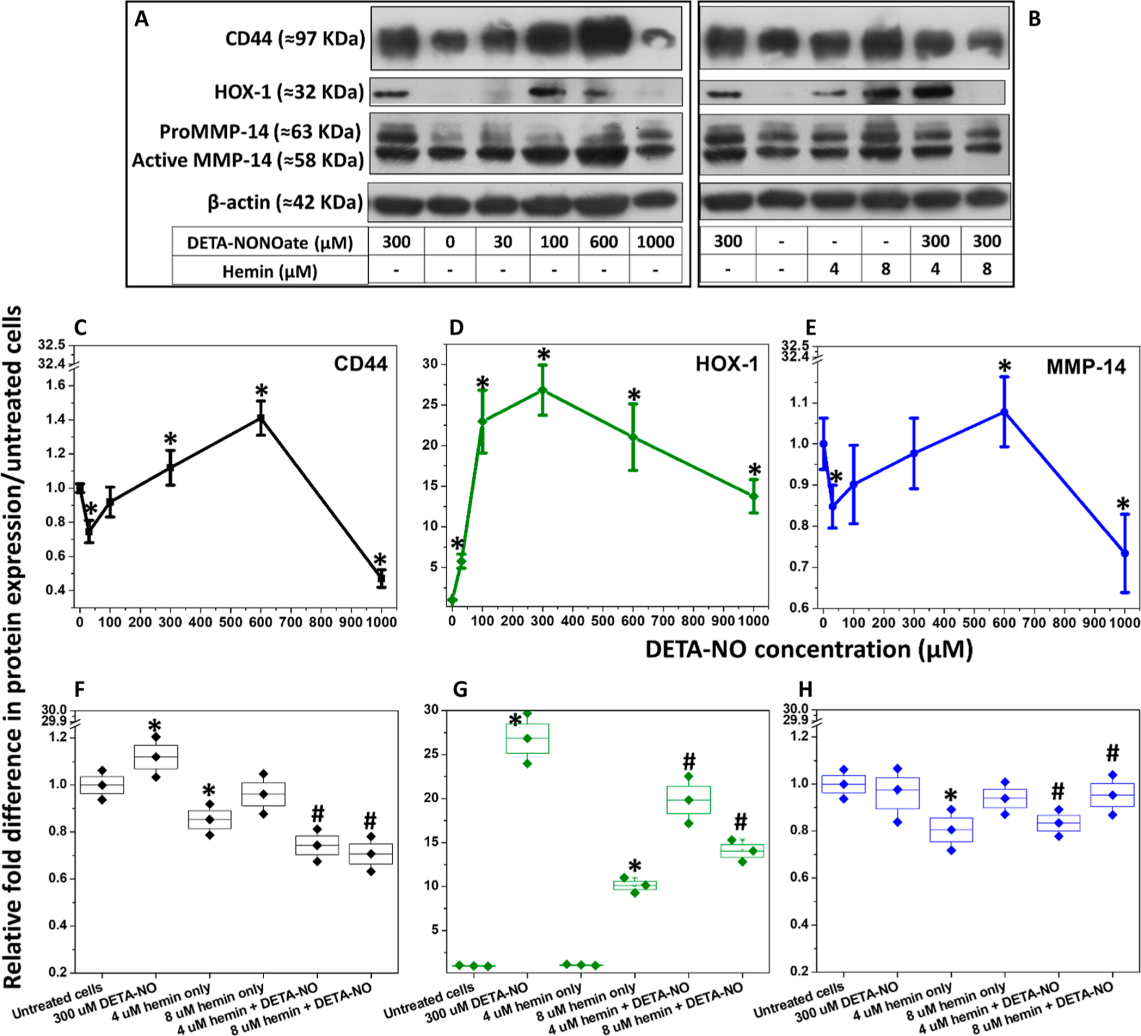
^•^NO and hemin cause changes in the expression of CD44, HOX-1, and MMP-14, in MDA-MB-231 cells. (A) Immunoblots with 0, 30, 100, 300, 600, and 1000 μM DETA-NONOate after cell treatment. (B) Immunoblots after cell treatment with 300 μM DETA-NO and/or 4 or 8 μM hemin utilizing 5 μg proteins/well, respectively. This was followed by relative quantification of CD44 (C,F), HOX-1 (D,G), and active MMP-14 (E,H) obtained from triplicate samples and normalized onto β-actin. Results are presented as mean ± S.D, *n* = 3. *^,^#*P* < 0.05 compared to the control group (untreated cells) and DETA-NO only-treated cells (no added hemin) using a two-tailed unpaired student *t*-test.

^•^NO enhanced the expression of CD44 in MDA-MB-231 cells, which was proportional to the DETA-NO concentration up to 600 μM, followed by a significant drop at 1000 μM (Figure 5A,C). However, when the cells were treated with 300 μM DETA-NO in combination with hemin, the ^•^NO-induced CD44 expression decreased significantly (Figure 5B,F). A higher significant level of expression was detected in 4 μM hemin-treated cells. In the absence of ^•^NO, hemin enhanced HOX-1 expression in MDA-MB-231 cells, particularly at 8 μM, which was improved when cells were concomitantly treated with DETA-NO (Figure 5G). This later enhancement in expression was less than that observed in DETA-NO-only-treated cells. Both the pro-MMP-14 and active form were observed following blotting (Figure 5B). Slight changes in protein expression were detected, following cell treatment with DETA-NO, with a maximum enhanced expression at 600 μM DETA-NO and increased intensity of the active MMP-14 form (Figure 5E). Moreover, cell treatment with hemin with/without DETA-NO did not affect the MMP14 expression (Figure 5H).

**Figure 6:**
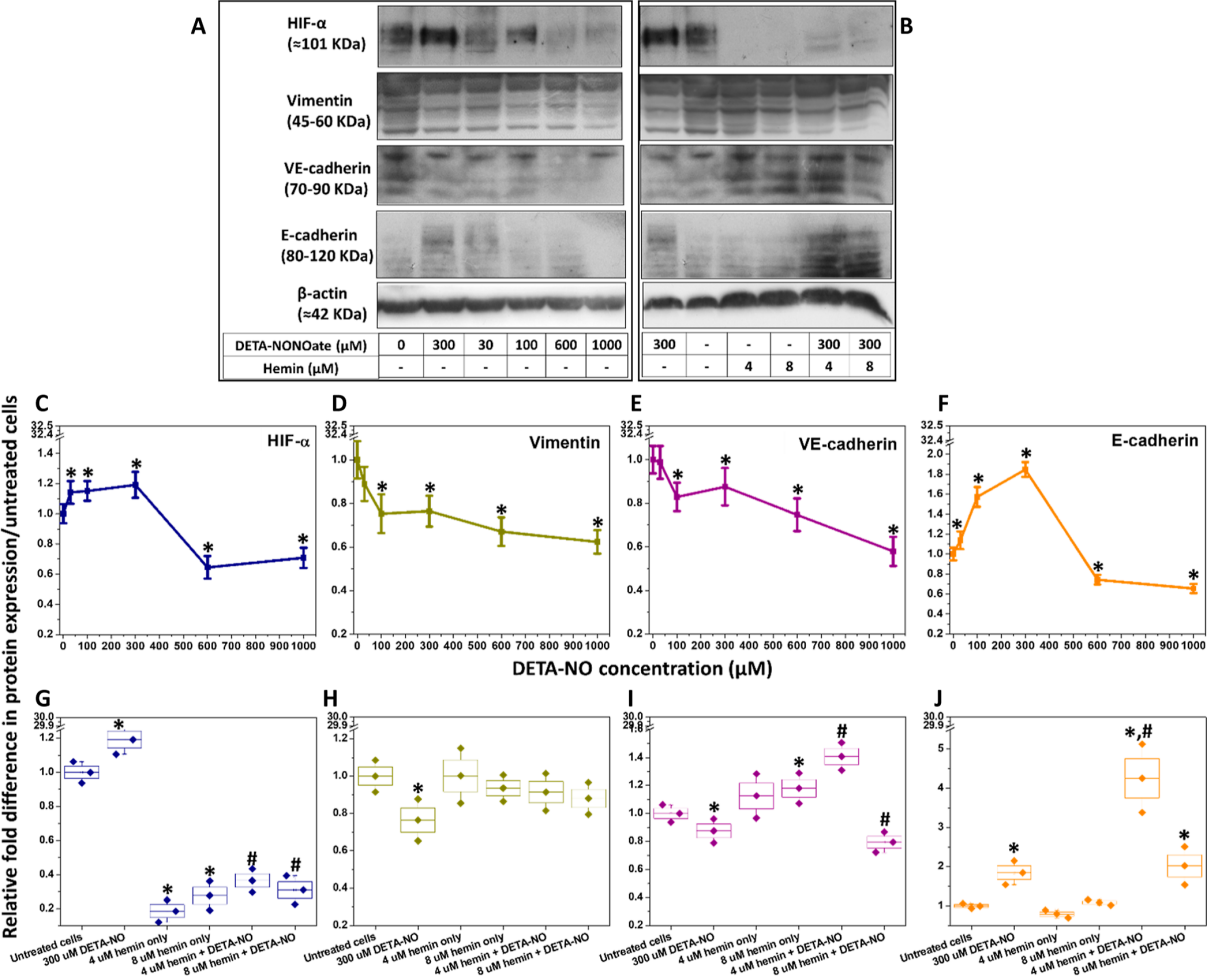
^•^NO and hemin cause changes in the expression of HIF-α, vimentin, VE-cadherin, and E-cadherin in MDA-MB-231 cells. (A) Immunoblots with 0, 30, 100, 300, 600, and 1000 μM DETA-NONOate after cell treatment. (B) Immunoblots after cell treatment with 300 μM DETA-NO and/or 4 or 8 μM hemin utilizing 20 μg proteins/well, respectively. This was followed by relative quantification of HIF-α (C,G), vimentin (D,H), VE-cadherin (E,I), and E-cadherin obtained from triplicate samples and normalized onto β-actin. Results are presented as mean ± S.D, *n* = 3. *^,^#*P* < 0.05 compared to the control group (untreated cells) and DETA-NO only-treated cells (no added hemin) using a two-tailed unpaired student *t*-test.

Figure 6A,C shows the change in the expression of HIF-α following cell treatment with different concentrations of DETA-NO. However, the cell treatment with hemin significantly inhibited HIF-α expression, irrespective of the exposure to ^•^NO (Figure 6B,G).

### ^•^NO, Hemin, and the Product of Their Reaction Regulate the Expression of Different EMT Markers

The different DETA-NO concentrations decreased the expression of vimentin (Figure 6D) and VE-cadherin (Figure 6E) gradually proportional to the concentration/flux of ^•^NO. This was accompanied by enhanced E-cadherin expression (Figure 6F), with a maximum accumulation in cells treated with 300 μM DETA-NO, while the higher concentrations reduced its levels.

The treatment of MDA-MB-231 cells with hemin did not change the expression of vimentin significantly compared to the untreated cells. Moreover, while DETA-NO decreased the vimentin expression, the treatment with the hemin/DETA-NO mixture restored the normal expression levels (Figure 6H). In addition, hemin, in the absence of ^•^NO, did not significantly change the expression of VE-cadherin (Figure 6I) and E-cadherin proteins (Figure 6J), which were enhanced in hemin/DETA-NO-treated cells. However, in the presence of ^•^NO, slight changes in the levels of VE-cadherin expression were found.

### ^•^NO Modulates the Mitochondrial Functions Dependent on Its Concentration and Flux

**Figure 7:**
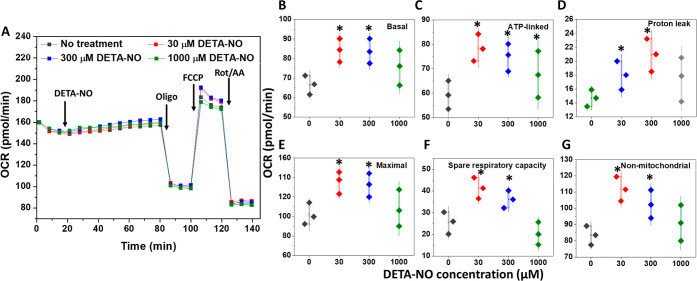
Impact of 1 h treatment with DETA-NO on the mitochondrial function of MDA-MB-231 cell measured by Mito Stress test. (A) Representative kinetic plot, which shows the changes in OCR values following the acute injection of 30, 300, and 1000 μM DETA-NO into cells and incubation for 1 h, followed by the normal procedures of the assay. The individual parameters of mitochondrial function were summarized as basal (B), ATP-linked (C), proton leak (D), maximal respiration (E), spare respiratory capacity (F) and non-mitochondrial-OCR (G). Data are represented as mean ± S.D; *n* = 3. *P < 0.05 compared to the untreated cells using a two-tailed unpaired student *t*-test. This experiment was repeated two times.

The effects of 30, 300, and 1000 μM DETA-NO concentrations on the mitochondrial functions were investigated following cell treatment for 1 and 24 h. A significant increase in the basal oxygen consumption rate (OCR) was observed after injecting 30 and 300 μM DETA-NO (Figure 7A). However, although the OCR values started to increase significantly directly after the injection of 1000 μM DETA-NO, these differences disappeared compared to cells treated with medium only by the last reading before oligomycin (Olig) injection (Figure 7B). However, the injection of Olig stimulated both ATP-linked OCR and proton leak significantly in cells treated with either 30 or 300 μM DETA-NO (Figure 7C,D). Similarly, 1000 μM DETA-NO showed similar effects but without significant differences from the untreated cells. Moreover, a higher but non-significant ATP-linked OCR was in the case of 30 μM DETA- NO, compared to the other concentrations, but this was accompanied with a lower proton leak in the former case.

The maximal respiration was estimated next after carbonyl cyanide 4-(trifluoromethoxy)phenylhydrazone (FCCP) injection, significantly enhancing the maximal OCR in cells treated with 30 and 300 μM DETA-NO. However, 1000 μM DETA-NO maintained maximal OCR levels similar to those of the untreated cells (Figure 7E). This was accompanied by an increase in the respiratory reserve capacity, which reached its highest levels at 30 μM DETA-NO, followed by 300 μM DETA-NO. In comparison, a decrease started in 1000 μM DETA-NO-treated cells (Figure 7F).

Similarly, an increase in the non-mitochondrial OCR was observed in 30 and 300 μM DETA-NO-treated cells, with a higher level at the former concentration and no significant changes in the case of 1000 μM DETA-NO (Figure 7G).

After 24 h of treatment, while the basal OCR increased at 30 μM DETA-NO compared to the untreated cells, the cell treatment with 300 μM DETA-NO caused its dropping (Figure S19A,B). The measured ATP-linked OCR in 30 μM DETA-NO-treated cells was higher than that of the untreated cells, indicating an increase in ATP demand, but with lower proton leak OCR (Figure S19C,D). In contrast, cell treatment with 300 μM DETA-NO decreased the ATP-linked OCR and proton leak. Furthermore, following FCCP injection, the maximal OCR and reserve capacity increased significantly in the case of 30 μM DETA-NO-treated cells, but decreased sharply with the other concentration (Figure S19E,F). Moreover, at the same time, cell treatment with 300 μM DETA-NO caused a significant drop in the non-mitochondrial OCR. There were no differences in the case of 30 μM DETA-NO (Figure S19G).

### Hemin Inhibits the Effects of ^•^NO on the Mitochondrial Functions in MDA-MB-231 Cells

**Figure 8:**
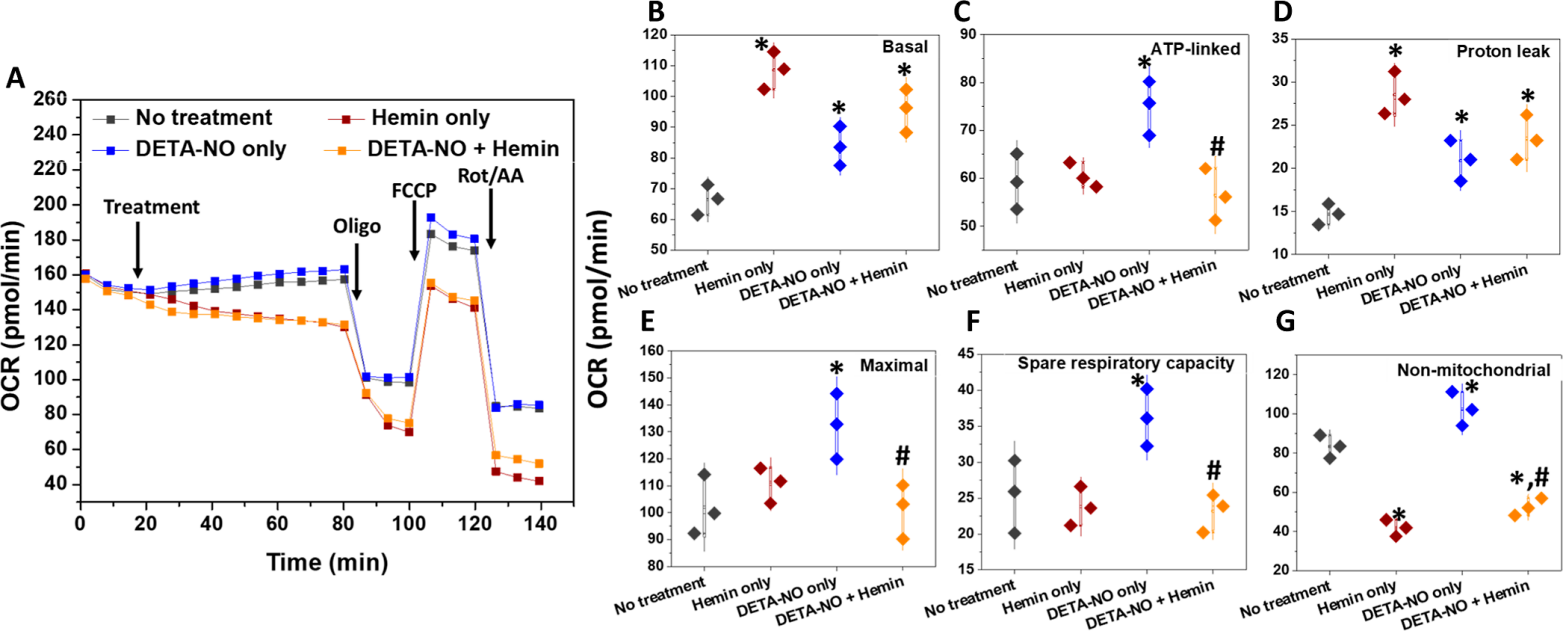
Impact of 1 h treatment with DETA-NO and/or hemin on the mitochondrial function of MDA-MB-231 cell measured by Mito Stress test. (A) Representative kinetic plot, showing the changes in OCR values following the acute injection of 300 μM DETA-NO and/or 8 μM hemin to cells and incubation for 1 h, followed by the normal procedures of the assay. The individual parameters of mitochondrial function were summarized as basal (B), ATP-linked (C), proton leak (D), maximal respiration (E), spare respiratory capacity (F), and non-mitochondrial-OCR (G). Results are presented as mean ± S.D, n = 3. *^,^#*P* < 0.05 compared to untreated and DETA-NO-only-treated cells using a two-tailed unpaired student *t*-test. This experiment was repeated two times.

The effects of hemin in the absence and presence of ^•^NO were evaluated. Generally, a decrease in the OCR and the extracellular acidification rate readings started once hemin was injected to the wells, referring to transient suppression of basal respiration (Figure 8A,B). The treatment of MDA-MB-231 cells with hemin for 1 h did not cause significant changes in both the maximal and ATP-linked OCR (Figure 8C,E), but induced an increased proton leak (Figure 8D). However, hemin-treatment for 24 h decreased the basal, maximal respiration and ATP production, but enhanced the proton leakage (Figure S20A–D). Furthermore, hemin did not affect the spare respiratory capacity whether added to cells in the presence or absence of ^•^NO for 1 h treatment (Figure 8F), which was inhibited after 24 h of culture with the different treatments (Figure S20E). However, this was accompanied by a significant drop in the non-mitochondrial OCR after incubation for one and 24 h (Figures 8G and S20F).

## Discussion

The study started with evaluation of the ^•^NO-release kinetics in different solutions. Generally, no significant differences in the degradation behavior of DETA-NO and ^•^NO-release kinetics were observed whether the electrochemical detection was performed in FBS-free or FBS-containing medium. However, the kinetics were higher in the buffer. Similar differences in the release kinetics from DETA-NO and other ^•^NO-donors in both tested solutions were reported before.^24^ However, while an enhancement in ^•^NO release was reported in a medium with 5% CO_2_ (cell culture incubator), this was not studied here due to the differences in the design of the ^•^NO-detection system. Moreover, the maximum release of ^•^NO from DETA-NO in Dulbecco’s modified Eagle medium (DMEM) in the incubator using the CellNO Trap device was around 10-fold, observed in the absence of 5% CO_2_.^24^ This difference was similar to that observed in our experiments in both buffer and medium. Hence, phosphate buffer is still a practical solution for testing the ^•^NO-release and scavenging in our current study; however, the pH was maintained at pH 7.4, not in the acidic solution as in the case of a medium with 5% CO_2_. Moreover, it should be mentioned here that the DETA-NO used in the whole study was from the same batch.

Previously, we reported how hemin interacts with ^•^NO released from DETA-NO in FBS-containing DMEM.^20^ Here, these interactions were investigated by UV–vis spectroscopy in phosphate buffer using a 16 μM hemin concentration for better resolution of the changes within the Soret band region. Interestingly, the effects of ^•^NO from 300 μM DETA-NO seem to have the most substantial impact on decreasing the A_382_ after 1 min. Moreover, from the UV–vis region 240–270, the DETA-NO concentration can be detected; the concentrations 300 and 1000 μM showed similar absorption at 252 nm, indicating a possible saturation of solution. This may explain the observed decrease in A_382,_ in Figure S2E.

There is still a debate on whether increasing or decreasing ^•^NO levels is the best strategy for breast cancer treatment.^25,26^ We reported before the effects of hemin, hemin derivatives, and ^•^NO on the migration of MDA-MB-231 and HCC1806 cells.^21^ The migration results demonstrated that ^•^NO-induced cell migration was inhibited in the presence of hemin, which was proportional to its concentration (Figures 1A and S5B). However, there were nearly equal effects of different DETA-NO concentrations on the cell invasion, with more significant effects of EGF than DETA-NO. It should be noted that no differences in the ^•^NO release from DETA-NO were observed in the presence and absence of collagen (data not shown). Moreover, as hemin did not affect the cell invasion in response to ^•^NO, this proposes the hindrance of ^•^NO-scavenging by hemin, mainly due to its interactions with collagen.

The effects of different concentrations of ^•^NO and/or hemin on MDA-MB-231 cells were studied by lectin staining. Collectively, these comparisons of results due to freshly prepared and degraded 300 μM DETA-NO indicate that the measured lectin-associated fluorescence are particular to the effects of ^•^NO released from DETA-NO, not from its degradation products, except for AAL lectin.

The alterations in the glycosylation patterns of several metastasis-associated glycoproteins in MDA-MB-231 cells were investigated previously.^4,5^ Following cell treatment with different DETA-NO concentrations, a differential carbohydrate expression was observed corresponding to certain glycosylation, fucosylation, and sialylation patterns of proteins. The enhanced expression of HPA-binding glycotopes in MCF-7 and MDA-MB-231 cells is one of the main factors promoting their invasive abilities.^5^ Moreover, lectin histochemical studies discovered a positive correlation between HPA-lectin binding and breast cancer metastasis.^27,28^

Our observed significant change in expression of PHA-L and HPA-binding glycoproteins indicates specific actions of ^•^NO concerning the nature of glycan epitope, the underlying enzymology and the cell line. Regarding the underlying enzymology, ^•^NO enhanced the activity of *N*-acetylglucosaminyl transferase I via ^•^NO/cGMP pathway toward the completion of terminal glycosylation of prolactin receptors in murine mammary epithelial cells.^29^ This is one of the essential enzymes involved in the N-linked glycosylation of different proteins,^30^ with an estimated 300 glycosyltransferases, produced in mammalian tissues.^31^ In contrast, the expression of this enzyme was reduced in response to enhanced iNOS expression and ^•^NO generation in mouse hepatocytes.^32^ These observations refer to different effects of ^•^NO on the glycosylation patterns, the expression, and/or activity of enzymes involved in this process. However, these comparisons cannot draw a direct conclusion, due to the different cellular systems, cell surface glycans, and the method of ^•^NO introduction.

As some of these reactions are non-enzymatic and proceed mainly via auto-oxidation of reducing sugars, ^•^NO could neutralize various radicals involved in this cascade and inhibit the formation of glycation products.^33^ On the other hand, considering the reactivity of ^•^NO as a free radical species, it was found to inhibit the glycoxidation reaction responsible for generating certain glycation end products. Hence, the ^•^NO flux up to those concentrations released from 300 μM DETA-NO may act as stimulator of the enzymatic pathways generating glycosylated proteins, which can be inhibited at higher ^•^NO/DETA-NO concentrations, due to the interference with the glycosylation and glycation pathways.

The surface fucosylation of MDA-MB-231 cells is correlated with their invasive capability, which was impaired via the defucosylation process with decreasing of the ECM-cells interactions.^34,35^ Moreover, the upregulation of fucosyltransferase 8 (FUT8), responsible for the attachment of fucose (α-1,6) to core GlcNAc of *N*-glycans in MDA-MB-231, promoted their migration and epithelial–mesenchymal transition (EMT).^36,37^ However, these effects were reversed with FUT8 knockdown. The observed differences in fluorescence due to UEA (Figures 3C and S9) and AAL-binding glycotopes (Figures 3D and S10) refer to increased levels of surface fucosylation, which was significant in the case of UEA-I-binding proteins at all DETA-NO concentrations.

The influence of ^•^NO on the sialylation of cellular proteins was also evaluated, due to the correlation between the upregulated sialyltransferases and promoted metastasis of different tumors.^38^ For instance, the enhanced expression of sialyltransferase, ST6Gal-I, responsible for the α-2,6-Sialylation, in MDA-MB-435 cells enhanced their adherence to collagen IV, reduced the cell–cell adhesion, and promoted their migration and invasion.^39^ Similarly, the upregulation and functionality of ST8SIA4, in the MDA-MB-231 cells, was closely examined, due to its roles in the malignant behavior of these cells.^40^ This proposed ST8SIA4 as a target for treating breast cancer progression. The observed enhancement in sialylation was also reported in MDA-MB cells, developed by in vivo selection of bone lesions following the intracardiac inoculation of MDA-MB-231 cells.^4^ This also correlated with the promoted activity of MDA-MB cells toward the selective colonization of bone and stimulating osteoclast differentiation.^41^

They support our findings regarding the enhancement of malignancy of MDA-MB-231 cells following DETA-NO treatment. This is dependent on concentrations of the released ^•^NO and its flux and correlated with the levels of cell surface sialylation. Our group demonstrated that abnormal glycosylation controls various facets of tumor biology, and it has long been recognized as a hallmark of cancer. Increased sialylated glycan expression has been seen in several forms of cancer, including multiple myeloma, and is frequently associated with aggressive metastatic behavior.^42^ Our results suggest that controlling flux of NO and its interactions with the breast cancer cells can be an approach for modulating of the levels of glycosylation, sialylation, and fucosylation toward the inhibition of their metastatic properties. This can be achieved by either the delivery of excessive amounts of NO or scavenging of NO, as explained in the next sections.

While hemin acts as a ^•^NO-scavenger, a mechanism similar to that observed following nitrite generation, entrapment of hemin within the protein moieties, and generation of H_2_O_2_, described before,^20^ may take place. This mechanism may be implicated towards enhanced terminal GalNAc and GlcNAc formation. Nevertheless, the excessive generation of reactive oxygen (ROS) and nitrogen species (RNS) in the case of DETA-NO/8 μM hemin can neutralize the carbonyl radicals involved in the glycation reaction as reported previously, preventing the formation of more N-linked glycans.^33^ However, this mechanism requires further examination, especially as the downstream effects of ^•^NO-scavenging were absent in the latter case.

These observations in the case of SNA (Figures 4E and S17) and MAA-binding glycotopes (Figures 4F and S18) relate mainly to the initial low fluorescence intensity corresponding to the SNA-binding glycotopes, making it difficult to compare the different groups. Moreover, the significant effects after cell treatment with DETA-NO and hemin relate to the enhanced uptake of hemin following its nitrosylation, as we reported before.^20^ These results, supported by the previous findings on the disruption of surface sialylation in hemin-treated erythrocyte^43^ and the inhibitory effects of upregulated HOX-1 in response to hemin,^44^ explain the decrease in fluorescence intensity in response to surface proteins with α-2,3-linked sialic acids. Hemin enhanced the expression of HOX-1, particularly in the presence of ^•^NO.

The impact of ^•^NO on the expression of proteins and enzymes associated with MDA-MB-231 cell migration was investigated first. CD44 is a marker of breast tumor metastasis, accompanied poor disease outcome.^45^ The ^•^NO-inducing effects for CD44 expression were reported before in MDA-MB-231, MDA-MB-468, MB-157, and Hs578T.^14^ These effects were suggested to be related to the enhanced nitrosative ^•^NO-signaling pathways, causing an increase in the basal-like phenotype.^46^ However, the drop in CD44 expression at 1000 μM DETA-NO most probably corresponds to the high ^•^NO flux reported to cause cell death with similar levels to those mediating the killing of pathogens and cancer types by the immune system.^47^ Similar effects of ^•^NO on the expression of HOX-1 were observed, with a maximum expression at 300 μM DETA-NO, followed by decreased levels at higher concentrations (Figure 5D). A similar response was reported in rat aortic smooth muscle cells, following the treatment with sodium nitroprusside, *S*-nitroso-*N*-acetyl-penicillamine, and the peroxynitrite donor, 3-morpholinosydnonimine.^48^ The ^•^NO effects were throwing both mRNA and protein levels of the enzyme responsible for heme/hemin degradation with generation of carbon monoxide (CO).

Although the decrease in CD44 expression in the presence of hemin and DETA-NO relate mainly to the ^•^NO-scavenging by hemin, there are still controversies regarding its exact effects on CD44 expression. On the one hand, there is a positive correlation between HOX-1 activity and CD44 expression with essential roles played in the self-renewal of human breast cancer stem cells.^49^ In addition, hemin is one of the main inducers of HOX-1 expression, causing heme/hemin degradation.^50,51^ This explains our findings, where, in the absence of ^•^NO, hemin enhanced HOX-1 expression in MDA-MB-231 cells (Figure 5G). Similar results were reported in MCF-7 breast cancer cells, accompanied with inhibition of the transforming growth factor-β1-induced EMT.^52^ On the other hand, the accompanied subsequent activity of HOX-1 helps maintain the expression of CD44, which explains the non-significant changes in CD44 in 8 μM hemin-treated cells (Figure 5F). However, when cells were treated with hemin and ^•^NO, multiple inducers with synergistic effects were expected to enhance HOX-1 expression. Although these effects were expected to be relatively higher in the case of 8 μM hemin than with the other hemin concentration, ^•^NO-scavenging seems to play a role, which was more significant with 8 μM hemin/DETA-NO mixture than with 4 μM hemin/DETA-NO. This explains the lower expression levels of HOX-1 in the former case due to the higher ^•^NO-scavenging efficiency and hemin nitrosylation.

Certain matrix metalloproteinases (MMPs) mediate cancer progression, with a number of ECM components controlling their expression and activity.^53^ MMP-14 is one of the essential MMPs expressed by the invasive TNBC cell lines, MDA-MB-231 and MDA-MB-436.^54^ In addition, the cross-talk between MMP-14 and CD44 is an essential step towards activating of cancer cell migration.^55^ Hence, the inhibition of MMP-14/CD44 hetero-dimerization was suggested as an efficient way to inhibit the MMP-14-mediated cancer cell migration.

A positive correlation was observed between the expression of both eNOS and MMP-14 in intratumoral vessels of human malignant melanomas^56^ and granulomatous lesions.^57^ Moreover, while ^•^NO is a positive regulator of the expression of MMP-9 in vascular endothelium^58^ and MMP-14 in murine macrophages,^59^ its effects on MMP-14 under the current experimental conditions were not significant.

In breast cancer, the overexpression of HIF-α is an independent predictor of poor patient prognosis, with different regulatory roles of cancer metastasis.^60^ However, there is still a debate on the ideal anti-cancer therapeutic approach for targeting HIF-α, and whether it should be targeting the blocking or enhancement of its expression and/or function.^61,62^ On the other hand, different physiological conditions can stabilize HIF-α, including the hypoxic environment within the tumor tissue,^63^ Co^2+^ ions, deferoxamine and certain ROS and RNS.^64^ Various studies reported the susceptibility of HIF-α expression and accumulation to certain ^•^NO concentrations. Most of these studies were performed under hypoxic conditions, where ^•^NO enhanced the degradation of HIF-α and decreased its accumulation.^65^ However, under normoxic conditions, ^•^NO stabilize the protein and enhances the HIF-α accumulation.^66^ This is mediated by the ^•^NO-inhibitory actions of prolyl hydroxylases (PHDs) activity under both the normoxic and hypoxic conditions, with its roles in the HIF-1α-prolyl hydroxylase-2 (PHD2) autoregulatory loop.^66^ However, in the case of hypoxia, and owing to the transient effects of ^•^NO only with low oxygen availability, an enhanced expression of PHD2 was observed at later stages of culture causing destabilization of HIF-α. Furthermore, as an independent mechanism to the ^•^NO/HIF-1α/PHD2 pathway, the ^•^NO-induced *S*-nitrosylation of HIF-α, particularly at cysteine 533 inhibited the protein degradation.^67^

The current study was performed under normal culture conditions and the observed increase in HIF-α protein expression up-to a DETA-NO concentration of 300 μM relates to the previously explained ^•^NO inhibitory actions of PHDs and the nitrosylation effects (Figure 6A,C). However, the decreased expression at 600 and 1000 μM DETA-NO can relate to the enhanced accumulation of HIF-α, due to the ^•^NO-induced inhibition of PHD2 at an earlier stage resulting in increased mRNA/protein expression of PHD2, which induces the HIF-α degradation. These effects may become more prominent at 600 and 1000 μM DETA-NO owing to the expected severe inhibition of PHD2 activity at the earlier stage of ^•^NO release, leading to further production and activation of PHD2 at later stages.^66^ Moreover, further nitrosylation/nitration effects of high concentrations ^•^NO can have different effects on the PHD2 activity and HIF-α degradation behavior, which needs further exploring.

Heat shock protein 90 (HSP90) upregulation was reported in various cancer cells as a response to different environmental stress conditions such as hypoxia.^68^ For instance, HSP90 regulates the expression of HIF-α, with inhibitory actions for its degradation in colon cancer cells,^69^ and breast cancer cell lines.^70^ However, hemin was found to inhibit the expression of client proteins of HSP90 causing a decrease in HIF-α accumulation.^69^ This was in combination with its interference with the CoCl_2_-induced HIF-α expression and the induction of protein degradation. These results explain our findings and confirm the same effects of hemin on different cancer cell lines. Moreover, hemin, following nitrosylation, maintained its activity against HIF-α stabilization.

During the EMT, the epithelial markers start to disappear gradually with the mesenchymal markers becoming prominent at the late stages of transition leading to a stable mesenchymal state.^71^ Vimentin filaments play vital roles in supporting the mechanical integrity of the migratory machinery for supporting the cancer cell migration and is overexpressed during EMT and cancer progression.^72^ There is a positive correlation between inhibiting of ^•^NO production and vimentin expression,^73^ with enhanced ^•^NO generation by vimentin (Vim^–/–^) macrophages compared to the WT types.^74^ Cadherins are a wide variety of cell adhesion molecules, which regulate the cancer cell migration with differential expressions during the EMT.^75^ Moreover, both the stabilization of vimentin and loss of the epithelial gene product E-cadherin have been reported as markers defining the mesenchymal phenotype of cells.^76^ E-cadherin is a type-I cadherin, while VE-cadherin is a type-II cadherin, and both of them play important roles in cancer cell interactions and tumor invasiveness.^77^

The findings in Figure 6D–F contradict the results of Switzer et al.,^46^ where the treatment of MDA-MB-468 with 500 μM DETA-NO enhanced vimentin expression and lowered that of E-cadherin. This effect may be due to the initial serum starvation of cells before the treatment with DETA-NO. At the same time, in our experiments, the culture medium was exchanged with FBS-free medium directly when DETA-NO was added to cells. Furthermore, it is noteworthy that the multiple bands detected in the case of vimentin relate to its different fragments isolated from cells.^78^ Moreover, MDA-MB-231 cells were reported to be E-cadherin-negative cell lines, while the expression of E-cadherin was observed in control MDA-MB-468.^79^ Accordingly, the irregular effects of ^•^NO on the E-cadherin-free cell line refer to the initial induction of the protein expression at the DETA-NO concentration within the range of 30–300 μM, followed by the regular inhibitory effects of higher concentrations of ^•^NO. Similar results to our findings were reported before, with testing the effects of 1000 μM DETA-NO only on vimentin expression in DU-145 and pC-3 prostate cancer cell lines.^80^ However, further investigations are ongoing in our lab to understand that topic.

For exploring the effects of ^•^NO and hemin on the mitochondrial functions of MDA-MB-231 cells, the mitochondrial stress assay was carried out using Olig, FCCP, and Rotenone/antimycin A (Rot/AA). The fluxes in OCR upon their sequential addition are used as an indicator of the mitochondrial (dys)function.^81^ Olig works as an inhibitor of ATP synthesis and FCCP is an uncoupling agent. Rot and AA are complex I and complex III inhibitors of the respiratory chain, respectively.

Generally, the deleterious effects of ROS and RNS target the mitochondrion and modulate the cellular bioenergetics.^82^ Generally, following Olig injection, the OCR values decrease due to the inhibition of ATP synthase, responsible for the oxidative phosphorylation of ADP to ATP and energy production. Hence, the remaining respiration relates to the protons pumped during electron transport resulting in oxygen consumption without ATP production.^83^ However, there is still a possibility that Olig would increase the mitochondrial membrane potential resulting in higher proton leak through the membrane, with a possible overestimation of the proton leak OCR,^84^ as observed here (Figure 7D).

Cytochrome *c* oxidase (complex IV) is responsible for the final transfer of electrons to oxygen,^85^ and, following the disruption of mitochondrial membrane potential by FCCP, the OCR by this complex reaches its maximum. Despite the reported inhibitory effects of ^•^NO for mitochondrial respiration at complex IV at comprehensive physiological levels (1–270 nM),^86,87^ these actions were absent under the current experimental conditions. Moreover, treating endothelial cells with different DETA-NO concentrations for 1 h decreased the maximal OCR and reserve capacity.^83^ Although these results contradict our findings, this relates to the different sensitivities of different cell lines towards ^•^NO concentration, duration of exposure, and/or the ^•^NO-donor. It can be concluded from these measurements that the continuous flux of ^•^NO affects the different mitochondrial functions, with a potential mitochondrial shutdown at the high concentrations of ^•^NO from 1000 μM DETA-NO.

Similar to its effects on complex IV, long-term exposure to ^•^NO was reported to inhibit complex I via *S*-nitrosylation^88,89^ and nitration of certain tyrosine residues.^90,91^ Moreover, ^•^NO proved its inhibitory effects for complex II by disrupting Fe–S complexes.^92^ Moreover, via its interactions with complex III, ^•^NO inhibits the functionality of this complex independent of oxygen concentration, with further inhibition of electron transfer.^93^ Despite these effects, the acute treatment of MDA-MB-231 cells with ^•^NO for 1 h did not induce inhibitory effects for the non-mitochondrial OCR.

Hemin was reported as a positive regulator for EMT and vimentin expression.^94^ These effects, in addition to the ^•^NO-scavenging by hemin, explain the restoration of the normal levels of vimentin expression in cells treated with hemin and DETA-NO. Considering the nature of MDA-MB-231 as an E-cadherin-negative cell line and MCF-7, as an E-cadherin-positive cell line,^79^ this can explain the observed results in the absence of ^•^NO. In addition, mediated by its inducing effects for HOX-1 expression and activity, hemin was found to inhibit EMT via increasing the expression of E-cadherin in MCF-7.^52^ This supports the observed enhancement of E-cadherin in the hemin/^•^NO-treated cells (Figure 6J) in response to the increased expression of HOX-1 (Figure 5H). However, another mechanism may be involved for enhancing the gene/protein expression of E-cadherin, depending on the combination of hemin and ^•^NO within the culture medium. One of these is the increased uptake of hemin in the presence of ^•^NO, as we reported previously,^20^ causing a promoted expression of HOX-1, and consequently E-cadherin.

The observed results following 24 h of treatment with DETA-NO indicate low ATP demand and possible severe oxidative phosphorylation damage, leading to lower OCR.^95^ Moreover, the downstream effects of ^•^NO, mainly through nitration of specific tyrosine residues in the β-subunit of complex V were reported to inhibit the enzyme activity and decrease the ATP generation rate.^95^

The decrease in maximal OCR after cell treatment with 300 μM DETA-NO confirms the previously reported inhibition of mitochondrial respiration by ^•^NO, mediated by the inhibition of cytochrome oxidase.^86,87^ Moreover, this confirms the sensitivity of MDA-MB-231 cells to the ^•^NO flux, where the continuous release of ^•^NO from 300 μM DETA-NO induced a decrease in the maximal OCR, compared to the results in case of acute response. Of note, this inhibition observed under prolonged time is caused by the *S*-nitrosation of specific cysteine residues in the enzyme protein structure^87^ as well as a decrease in the protein levels/expression.^83^ These observations, as explained before, relate to the inhibitory effects of the “continuously produced” ^•^NO in the medium for complex I, which supports the previous findings.^88−91,93^ To summarize these findings, comparing of the OCR results in the case of 300 μM DETA-NO treatment for 1 and 24 h confirms the sensitivity of the respiratory chain in MDA-MB-231 cells to the continuous flux of ^•^NO. However, the low concentration of ^•^NO released from 30 μM DETA-NO generally activated mitochondrial respiration. These results help explain the roles played by ^•^NO on modulation of the mitochondrial metabolic outputs, particularly due to the reported roles of mitochondria in the invasion and motility of cancer cells.^96,97^

Similar effects of hemin on mitochondrial respiration in endothelial cells were reported as hemin-induced mitochondrial toxicity.^98^ However, following hemin treatment for 1 h, the basal OCR was significantly higher compared to the untreated cells as well as DETA-NO only treated cells. Reflected by its inducing effects for HOX-1 expression and activity, hemin at concentrations higher than 2 μM was reported to decrease the maximal respiration and ATP production and increased proton leakage in murine embryonic fibroblasts.^99^ However, the hemin treatment of retinal microvascular endothelial cells, with inhibited ferrochelatase, enhanced the expression and activity of cytochrome c oxidase, besides restoring normal mitochondrial respiration.^100^ Ferrochelatase is responsible for the final stage of mitochondrial heme generation via the insertion of Fe(II) into the PPIX. In our study, the cell treatment with hemin for 1 h did not change the maximal and ATP-linked OCR significantly (Figure 8C,E), which can relate to the short exposure period to hemin. Moreover, hemin cancelled the ^•^NO-induced OCR increase. Nevertheless, hemin increased the proton leak (Figure 8D), indicating some inhibitory effects for olig activity, with a possible decrease in the mitochondrial membrane potential. Moreover, the effects of the hemin/DETA-NO mixture were higher than that of DETA-NO only but less than the OCR levels in hemin-only-treated cells, indicating less effects of hemin/^•^NO mixture on the integrity of membrane potential.

Similar findings to the effects of hemin after 24 h of cell treatment were reported before.^99^ Hemin injection and incubation for 2.5 h with cells before olig injection started to cause a decrease in the basal and ATP-linked-respiration, and more increase in the proton leak. This was accompanied by similar effects of hemin incubated for one and 2.5 h on the maximal, spare respiratory capacity and non-mitochondrial respiration (data not shown). These results refer to similar activity of hemin and hemin/^•^NO mixture on the mitochondrial functions in cells, which are more prominent when tested within short period. However, the prolonged cellular exposure to ^•^NO, in combination with its enhancing effects for the cellular uptake of hemin causes significant changes in the influence of hemin on mitochondrial respiration. Generally, these aspects should be taken into consideration while developing certain therapeutics for TNBC.

## Conclusions

As one of the main cellular gasotransmitters, the excessive production of ^•^NO within the TNBC tissue has implications for tumor size growth and the corresponding blood supply dependent on its flux and concentration. Hence, hemin was proposed as a potential ^•^NO-scavenging compound and its potency has been evaluated toward the inhibition of TNBC cancer cell migration.

In this paper, we first studied the interactions between hemin and ^•^NO using UV–vis spectroscopy, and these results confirm our previous observations on how hemin quenches the high levels of ^•^NO. Next, the effects of different concentrations of ^•^NO on the migration of MDA-MB-231 cells as a model TNBC cell line were studied alongside how hemin modulates that by ^•^NO-scavenging. The ^•^NO-induced cell migration depended on the concentration of DETA-NO and the accompanied ^•^NO-flux, with a maximum enhancement in cells treated with 300 μM of the ^•^NO-donor. These observations were supported by promoted expression of CD44 and cell surface glycoproteins, in terms of hyper-glycosylation, sialylation, and fucosylation, particularly at DETA-NO concentrations higher than 100 μM up-to 600 μM. However, hemin treatment inhibited the ^•^NO-induced cell migration alongside interfering with the associated dysregulated cell surface glycoprotein expression. Moreover, certain ^•^NO concentrations modulated the expression of some proteins and enzymes involved in cancer cell migration, including, MMP-14, HOX-1, HIF-α as well as EMT-corresponding markers. However, hemin contradicted some of these signals, including the inhibition of ^•^NO-induced CD44, MMP-14, and HIF-α expression with slight effects on the EMT-related markers. Interestingly, some of these mechanistic effects relate to hemin itself, while others depend on its product after binding with ^•^NO. Finally, the influence of both ^•^NO and hemin on the mitochondrial functions depended on the period of exposure, with distinctive effects of each of them as well as following hemin nitrosylation. The reported results help understand how hemin modulates the effects of ^•^NO on MDA-MB-231 and is considered an important step towards developing new therapeutics for TNBC. However, its influence on the other cells residing within the tumor tissue, including the endothelial cells and immune cells, and on the interactions between them was out of the scope of the current work. These approaches and the possible combination of hemin or one of its derivatives with other breast cancer therapeutics toward a more effective treatment approach for TNBC are currently being investigated, by comparing their effects to those of aminoguanidine on iNOS-transfected TNBC cells. These studies were out of the scope of the current study.

## Experimental Section

### Cell Line and Reagents

MDA-MB-231 cells (HTB-26) were from the American Type Culture Collection. Hemin, NaH_2_PO_4_·2H_2_O, Na_2_HPO_4_·2H_2_O, anhydrous dimethyl sulfoxide (DMSO), paraformaldehyde (PFA), 4′,6-diamidino-2-phenylindole dihydrochloride, 2-(4-amidinophenyl)-6-indolecarbamidine dihydrochloride (DAPI), periodic acid, RPMI-1640 medium, DMEM, l-glutamine, penicillin/streptomycin, FBS, and phosphate-buffered saline (PBS) were purchased from Sigma-Aldrich. The tris buffered saline (TBS) components, including Tris-base, KCl, NaCl, CaCl_2_, and MgCl_2_, were all from Sigma-Aldrich. Recombinant human epidermal growth factor (EGF) and Tween 20 were obtained from Fisher Scientific. Bovine collagen type I was purchased from BD Biosciences. The ^•^NO-donors DETA-NO and *S*-nitroso-*N*-acetyl-penicillamine (SNAP) were from Cayman Chemicals. The transwell inserts (membrane 8.0 μm pores) were from Cruinn Diagnostics. The FITC-labeled AAL, UEA, HPA, and PHA-L and TRITC-labeled SNA and MAA lectins were purchased from EY Laboratories, Inc. The biotinylated AAL (B-1395-1), UEA (B-1065-2), SNA (B-1305-2) and MAA (B-1315-2) were from Vector Laboratories. Avidin and biotinylated HRP were from Vector laboratories. The antibodies for CD44 (MA5-15462), HOX-1 (MA1-112), iNOS (MA5-17139), MMP-14 (MA5-32076) and the secondary antibodies were obtained from Invitrogen. The antibodies for E-cadherin (ab40772), VE-cadherin (ab166715), vimentin (ab8978), and HIF-α (ab179483) were from Abcam, while beta-actin (β-actin) antibody (A5441) was from Sigma-Aldrich. The μ-Plate 96 Well Black ibiTreat #1.5 polymer coverslip was from IBIDI GMBH.

### ^•^NO-Procedures

The ^•^NO release from different concentrations of DETA-NO in FBS-containing DMEM and phosphate buffer (50 mM, pH 7.4) was measured as described in detail before.^20^ In brief, the ^•^NO release profile was measured electrochemically using a TBR 1025 Free Radical Analyser and an ISO-NOP007 micro-sensor [World Precision Instruments (WPI) Ltd, USA]. A stock solution of DETA-NO was prepared in 0.01 M NaOH. For each measurement, the stock was thawed for 5 min before injecting it into the testing solution containing the pre-polarized micro-sensor to reach a specific final concentration, with the voltage recording. From the SNAP-based standard curve, the voltage readings were employed for detecting the concentration of ^•^NO released over time in solution.

### UV–Vis Study

A stock solution of hemin was first prepared in DMSO. Then, the UV–vis spectrum of 16 μM hemin diluted in phosphate buffer (50 mM, pH 7.4) was recorded in a quartz cuvette using a Biologic MOS-500 spectrometer. This was followed by recording the spectra each minute for 10 min. Next, DETA-NO was injected into the solution for different final concentrations of 30, 100, 300, or 1000 μM.

### In Vitro Study

#### Cell Migration

The effects of different concentrations of ^•^NO donor DETA-NO and hemin, on MDA-MB-231 cell migration were measured as previously described.^21^ The cells migrated for 24 h were detected by DAPI staining and fluorescence microscopy using an Olympus IX81 Inverted Fluorescence Phase Contrast microscope. For invasion assessment, the insert was coated overnight with 20 μg/mL collagen type I, washed with PBS, and then seeded with cells as described for in migration assay. 10 ng/mL EGF was employed as the positive control.

#### Lectin Staining

The cells were seeded at a density of 3 × 10^4^ cells per each well of μ-Plate 96 Well Black, cultured in 10% FBS-containing RPMI medium, and supplemented with 2 mM l-glutamine for 24 h at 37 °C in 5% CO_2_. The media were then exchanged with FBS-free medium containing different concentrations of DETA-NO. For evaluating the effects of hemin, it was diluted in the media. It was either added directly to the cells, or mixed further with DETA-NO-containing medium before adding to them (*n* = 3 for each group). The final tested concentrations of hemin were 4 and 8 μM. For comparison, a control group containing cells treated with 300 μM degraded DETA-NO, prepared by incubation at 37 °C for 3 months, was employed. Following 24 h of cell incubation, all wells were washed with PBS, fixed in 4% PFA for 15 min, and then washed with Tris-buffered saline for lectins (pH 7.2, TBSL). This solution contained 20 mM tris base, 100 mM NaCl, 1 mM CaCl_2_, and 1 mM MgCl_2_. The lectin staining procedures were then performed. In brief, after blocking with 2% periodate-pretreated BSA in TBS for 1 h at room temperature, the cells were washed and incubated overnight with a fluorescently labeled lectin in TBSL containing 0.05% tween20 (TBSL-T) at 4 °C. These were FITC-labeled AAL, UEA, HPA, and PHA-L and TRITC-labeled SNA and MAA, with a final optimized concentration of 20 μg/mL. This was followed by cell washing with TBSL-T, incubation with DAPI for 5 min at RT and final washing with TBSL. The cells were finally imaged using the Operetta high-content imaging system (PerkinElmer, Waltham, MA). FITC was excited at 480 nm and detected using the filter for Alexa Fluor 488, while TRITC was excited at 535 nm.

#### Western Blotting

4 × 10^5^ cells were cultured in FBS-containing RPMI-1640 medium in T-75 flasks and left for 24 h at 37 °C in 5% CO_2_ for attachment and reaching a confluency of approximately 80%. Next, the medium was exchanged with an FBS-free RPMI-1640 medium containing either one of the concentrations of DETA-NO, 4 or 8 μM hemin, or a mixture of 300 μM DETA-NO and 4 or 8 μM hemin. A control group containing cells treated with 300 μM degraded DETA-NO was employed for results comparison. The cells were cultured for 24 h at 37 °C in 5% CO_2_. Next, the cellular proteins were extracted as we detailed before.^20^ The proteins were resolved using 10% sodium dodecyl sulfate-polyacrylamide gel electrophoresis (SDS-PAGE). For western blotting, 5 μg proteins were loaded per well for the further detection of CD44, HOX-1, iNOS, and MMP-14 proteins and 20 μg proteins for detecting HIF-α, vimentin, E-cadherin, and VE-cadherin proteins. Following SDS-PAGE, the proteins were transferred to a nitrocellulose membrane for a 1 h blocking in 5% skimmed milk in 1× Tris-buffered saline (pH 7.6, TBS) with 0.05% tween20 (TBS-T) at room temperature. This was followed by overnight incubation with the primary antibody in 5% skimmed milk at 4 °C. The TBS for western blotting had the same composition as TBSL without CaCl_2_ and MgCl_2_. After washing membranes, they were probed with horseradish peroxidase (HRP)-conjugated goat anti-mouse or anti-rabbit secondary antibody for 1 h at room temperature. The proteins were finally detected and visualized using the Thermo Scientific SuperSignal West Pico PLUS Chemiluminescent Substrate. Next, the membranes were mildly stripped and probed with a mouse monoclonal β-actin antibody (1: 10,000), followed by the HRP-conjugated secondary antibody. The blotting was performed through three independent experiments with testing two samples per group.

The same general procedures as in western blotting were followed for lectin blotting, with some changes. 5 μg proteins were loaded per well, and the membranes were first blocked as described for probing with mouse monoclonal β-actin antibody (1: 10,000). Next, after mild stripping, the membranes were washed two times with TBSL-T, then blocked in 5% BSA in TBSL-T for 1 h at room temperature. This was followed by overnight incubation with biotinylated UEA (0.5 μg/mL), AAL (0.5 μg/mL), SNA (0.5 μg/mL), or MAA (1 μg/mL) at 4 °C. Next, all membranes were washed with TBSL-T and incubated with premixed avidin and biotinylated HRP in TBSL-T for 1 h at room temperature. The proteins were finally detected, as in the case of western blotting.

#### Real-Time Measurement of Mitochondrial Functions

The Cell Mito Stress Test was employed for evaluating the mitochondrial functions via the measurement of OCR of cells in real time using XFp Extracellular Flux Analyzer (Seahorse Bioscience, Agilent technologies, U.K). MDA-MB-231 cells were seeded in XFp Analyzer Cell Culture mini plates at a density of 2 × 10^4^ cells/well and left to attach overnight at 37 °C in 5% CO_2_ with reaching a confluency of nearly 80%. Cells were then washed with unbuffered Agilent Seahorse XF Base Medium (DMEM) and incubated for 1 h at 37 °C without % CO_2_. The latter medium was prepared freshly and contained 1 mM sodium pyruvate, 10 mM glucose, and 2 mM glutamine, and the pH was adjusted to 7.4. Hemin and DETA-NO were diluted freshly in the same medium just before the main assay procedures. The Mito Stress assay started with recording of the basal OCR, followed by injection of hemin and/or DETA-NO into the cells, with a continuous recording of the OCR for 60 min. Next, Olig, FCCP, and Rot/AA were sequentially injected, with a final concentration of 1 μM for each in every well. The injections were accompanied by recording of the changes in the OCR values, corresponding to the sequential changes in bioenergetics. Three independent experiments calculated parameters such as basal, ATP-linked, and reserve capacity OCR from the Mito Stress assays.

### Statistical Analysis

The results were statistically analyzed using the SPSS Computer program (version: 26). All data were expressed as the means ± S.D and were analyzed using *t*-test or one-way ANOVA, and the differences were considered statistically significant at (*P* < 0.05).

## Supporting Information Available

Change in fluorescence intensity corresponding to binding of the lectins PHA-L, HPA, UEA-L, AAL, SNA, and MAA with surface proteins before and after treatment of MDA-MB-231 cells with freshly prepared 300 μM DETA-NO or 300 μM DETA-NO degraded via incubation for 3 months at 37 °C; temporal changes in the voltage signal recorded over 2000 s; ^•^NO flux depends on DETA-NO concentration and decreases the intensity of the Soret band of hemin; temporal changes in the voltage signal recorded over 120 min following the injection of 30, 100, 300, 600, and 1000 μM DETA-NO only into FBS-containing medium and phosphate buffer; difference UV–vis spectrum of hemin following titration against 30, 100, 300, and 1000 μM DETA-NO in phosphate buffer over 10 min; Box-whisker blots showing the distribution of the number of counted cells migrated through the transwell membranes towards the chemoattractant composed of FBS-containing RPMI; temporal changes in the voltage signal and NO concentration recorded over 110 min following the injection of fresh and degraded DETA-NO into phosphate buffer with a final concentration of 300 μM; effects of DETA-NO on the expression of PHA-L-binding proteins; effects of DETA-NO on the expression of HPA-binding proteins; effects of DETA-NO on the expression of UEA-I-binding proteins; effects of DETA-NO on the expression of AAL-binding proteins; effects of DETA-NO on the expression of SNA-binding proteins; effects of DETA-NO on the expression of SNA-binding proteins; effects of hemin and DETA-NO on the expression of PHA-L-binding proteins; effects of hemin and DETA-NO on the expression of HPA-binding proteins; effects of hemin and DETA-NO on the expression of UEA-I-binding proteins; effects of hemin and DETA-NO on the expression of AAL-binding proteins; effects of hemin and DETA-NO on the expression of SNA-binding proteins; effects of hemin and DETA-NO on the expression of SNA-binding proteins; the impact of 24 h treatment with DETA-NO on the mitochondrial function of MDA-MB-231 cell measured by Mito Stress; and the impact of 24 h treatment with DETA-NO and/or hemin on the mitochondrial function of MDA-MB-231 cell measured by Mito Stress test (PDF)

## Abbreviations

AAL: *Aleuria aurantia* Lectin
CD44: cluster of differentiation 44
CO: carbon monoxide
DAPI: 4′,6-diamidino-2-phenylindole dihydrochloride, 2-(4-amidinophenyl)-6-indolecarbamidine dihydrochloride
DETA-NO: diethylenetriamine NONOate
DMEM: Dulbecco’s modified Eagle medium
DMSO: dimethyl sulfoxide
E-cadherin: epithelial cadherin
EGF: epidermal growth factor
EMT: epithelial–mesenchymal transition
FBS: fetal bovine serum
FCCP: carbonyl cyanide 4-(trifluoromethoxy)phenylhydrazone
FUT8: fucosyltransferase 8
HIF-α: hypoxia-inducible factor 1-alpha
HOX-1: heme-oxygenase
HPA: *Helix pomatia* agglutinin
HRP: horseradish peroxidase
HSP90: heat shock protein 90
iNOS: inducible nitric oxide synthase
MAA: *Maackia amurensis* lectin I
MMP-14: matrix metallopeptidase 14
OCR: oxygen consumption rate
Olig: oligomycin
PBS: phosphate-buffered saline
PFA: paraformaldehyde
PHA-L: *Phaseolus vulgaris* leucoagglutinin
PHD: prolyl hydroxylase
RNS: reactive nitrogen species
ROS: reactive oxygen species
Rot/AA: rotenone/antimycin A
SDS-PAGE: sodium dodecyl sulfate-polyacrylamide gel electrophoresis
SNA: *Sambucus nigra* lectin
SNAP: *s*-nitroso-*n*-acetyl-*d*,l-penicillamine
TBS: tris buffered saline
TNBC: triple-negative breast cancer
UEA: *Ulex europaeus* agglutinin I
UV–vis: ultraviolet–visible
VE-cadherin: vascular endothelial–cadherin
