# Supplementary material for: Nitric Oxide-Scavenging, Anti-Migration Effects, and Glycosylation Changes after Hemin Treatment of Human Triple-Negative Breast Cancer Cells: A Mechanistic Study
Source: ACS Pharmacol Transl Sci. 2023 Sep 11;6(10):1416–32. doi: 10.1021/acsptsci.3c00115 (PMC10580390; doi:10.1021/acsptsci.3c00115)
Supplement: Supplementary file 1 — pt3c00115_si_001.pdf [file pt3c00115_si_001.pdf]

Supporting information

Nitric Oxide-Scavenging, Anti-Migration Effects and Glycosylation Changes after Hemin Treatment of Human Triple-Negative Breast Cancer Cells: A Mechanistic Study

Amir M. Alsharabasy,[1] Amal Aljaabary,[1] Raghvendra Bohara,[1] Pau Farràs,[1,2] Sharon A. Glynn,[1,3] Abhay Pandit[1*]

[1]CÚRAM, SFI Research Centre for Medical Devices, University of Galway, Ireland. Postal code: H91 W2TY.
[2]School of Biological and Chemical Sciences, Ryan Institute, University of Galway, Ireland. Postal code: H91 TK33.
[3]Discipline of Pathology, Lambe Institute for Translational Research, School of Medicine, University of Galway, Ireland. Postal code: H91 YR71.

*AP: E-mail: Abhay.pandit@universityofgalway.ie

TABLE OF CONTENT

List of table captions/legends

Supplementary Table S1. The change in fluorescence intensity corresponding to binding of the lectins PHA-L, HPA, UEA-L, AAL, SNA and MAA with surface proteins before and after treatment of MDA-MB-231 cells with freshly prepared 300 µM DETA-NO or 300 µM DETA-NO degraded via incubation for 3 months at 37°C...............**S3**

List of figure captions/legends

Supplementary Figure S1. Temporal changes in the voltage signal recorded over 2000 sec following the injection of 30 (dark cyan colour) and 300 µM (blue colour) into FBS-containing medium (solid lines) and FBS-free medium (dashed lines)..........**S3**

Supplementary Figure S2. •NO flux depends on DETA-NO concentration, and decreases the intensity of the Soret band of hemin following binding..........**S4**

Supplementary Figure S3. Temporal changes in the voltage signal recorded over 120 min following the injection of 30 (dark cyan, i), 100 (red colour, ii), 300 (blue colour, iii), 600 (magenta colour, iii), and 1000 µM (olive colour, v) DETA-NO only into FBS-containing medium **(A)** and phosphate buffer (50 mM, pH 7.4) **(B)**....**S5**

Supplementary Figure S4. Difference UV-vis spectrum of hemin following titration against 30 **(A),** 100 **(B),** 300 **(C),** and 1000 µM **(D)** of DETA-NO in phosphate buffer (50 mM, pH 7.4) over ten minutes. **S6**

Supplementary Figure S5. Box-whisker blots showing the distribution of the number of counted cells migrated through the transwell membranes towards the chemoattractant composed of FBS-containing RPMI.....................**S7**

Supplementary Figure S6. Temporal changes in the voltage signal (A) and NO concentration (B) recorded over 110 min following the injection of fresh (blue colour) and degraded DETA-NO (orange colour) into phosphate buffer (50 mM, pH 7.4) with a final concentration of 300 μM..**S8**

Supplementary Figure S7. Effects of DETA-NO on the expression of PHA-L-binding proteins.............................**S9**

Supplementary Figure S8. Effects of DETA-NO on the expression of HPA-binding proteins...............................**S10**

Supplementary Figure S9. Effects of DETA-NO on the expression of UEA-I-binding proteins............................**S11**

Supplementary Figure S10. Effects of DETA-NO on the expression of AAL-binding proteins..............................**S12**

Supplementary Figure S11. Effects of DETA-NO on the expression of SNA-binding proteins...............................**S13**

Supplementary Figure S12. Effects of DETA-NO on the expression of SNA-binding proteins..............................**S14**

Supplementary Figure S13. Effects of hemin and DETA-NO on the expression of PHA-L-binding proteins.......**S15**

Supplementary Figure S14. Effects of hemin and DETA-NO on the expression of HPA-binding proteins..........**S17**

Supplementary Figure S15. Effects of hemin and DETA-NO on the expression of UEA-I-binding proteins........**S19**

Supplementary Figure S16. Effects of hemin and DETA-NO on the expression of AAL-binding proteins...........**S21**

Supplementary Figure S17. Effects of hemin and DETA-NO on the expression of SNA-binding proteins...........**S23**

Supplementary Figure S18. Effects of hemin and DETA-NO on the expression of SNA-binding proteins..........**S25**

Supplementary Figure S19. The impact of 24-hour treatment with DETA-NO on the mitochondrial function of MDA-MB-231 cell measured by Mito Stress test..**S26**

Supplementary Figure S20. The impact of 24-hour treatment with DETA-NO and/or hemin on the mitochondrial function of MDA-MB-231 cell measured by Mito Stress test..**S27**

Supplementary Table S1. The change in fluorescence intensity corresponding to binding of the lectins PHA-L, HPA, UEA-L, AAL, SNA and MAA with surface proteins before and after treatment of MDA-MB-231 cells with freshly prepared 300 µM DETA-NO or 300 µM DETA-NO degraded via incubation for 3 months at 37°C. Results are presented as mean ± S.D, n = 3. *, P < 0.05 versus the untreated cells using a two-tailed unpaired student t-test.

Lectin	No treatment	300 µM DETA-NO	300 µM degraded DETA-NO
PHA-L	17.4 ± 0.16	23.93 ± 1.11*	18.02 ± 1.86
HPA	15.36 ± 1.22	24.65 ± 3.66*	14.96 ± 0.5
UEA-L	7.99 ± 0.17	12.29 ± 0.46*	7.14 ± 0.2
AAL	24.98 ± 1.12	27.67 ± 6.53	7.77 ± 1.39*
SNA	1.1 ± 0.24	1.42 ± 0.34	1.28 ± 0.59
MAA	4.45 ± 0.78	7.02 ± 0.26*	4.88 ± 0.1

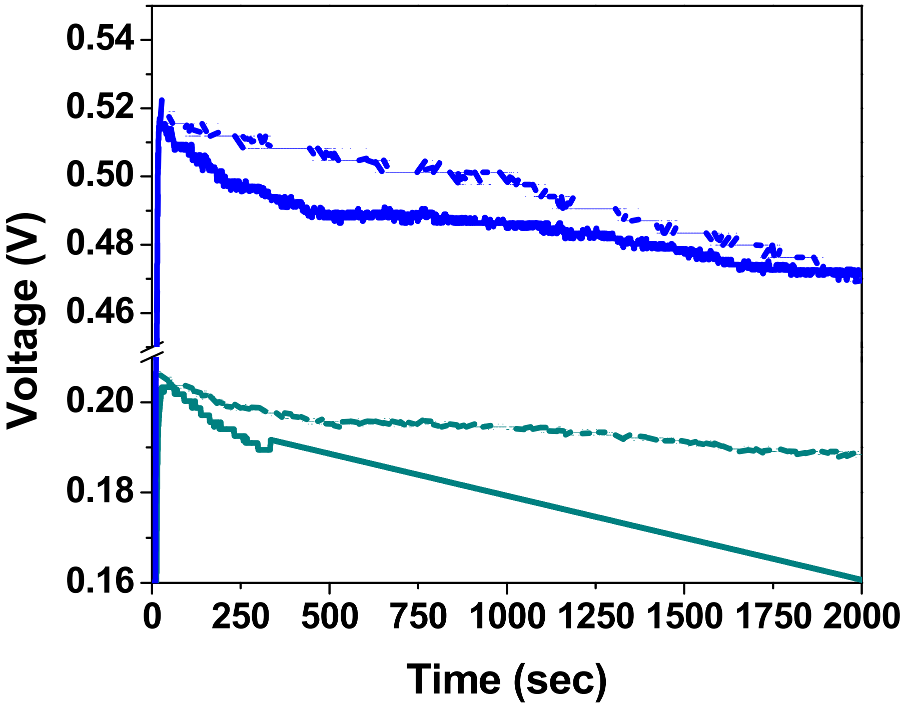

Supplementary Figure S1. Temporal changes in the voltage signal recorded over 2000 sec following the injection of 30 (dark cyan colour) and 300 µM (blue colour) into FBS-containing medium (solid lines) and FBS-free medium (dashed lines). Results are presented as mean voltage readings, n = 3.

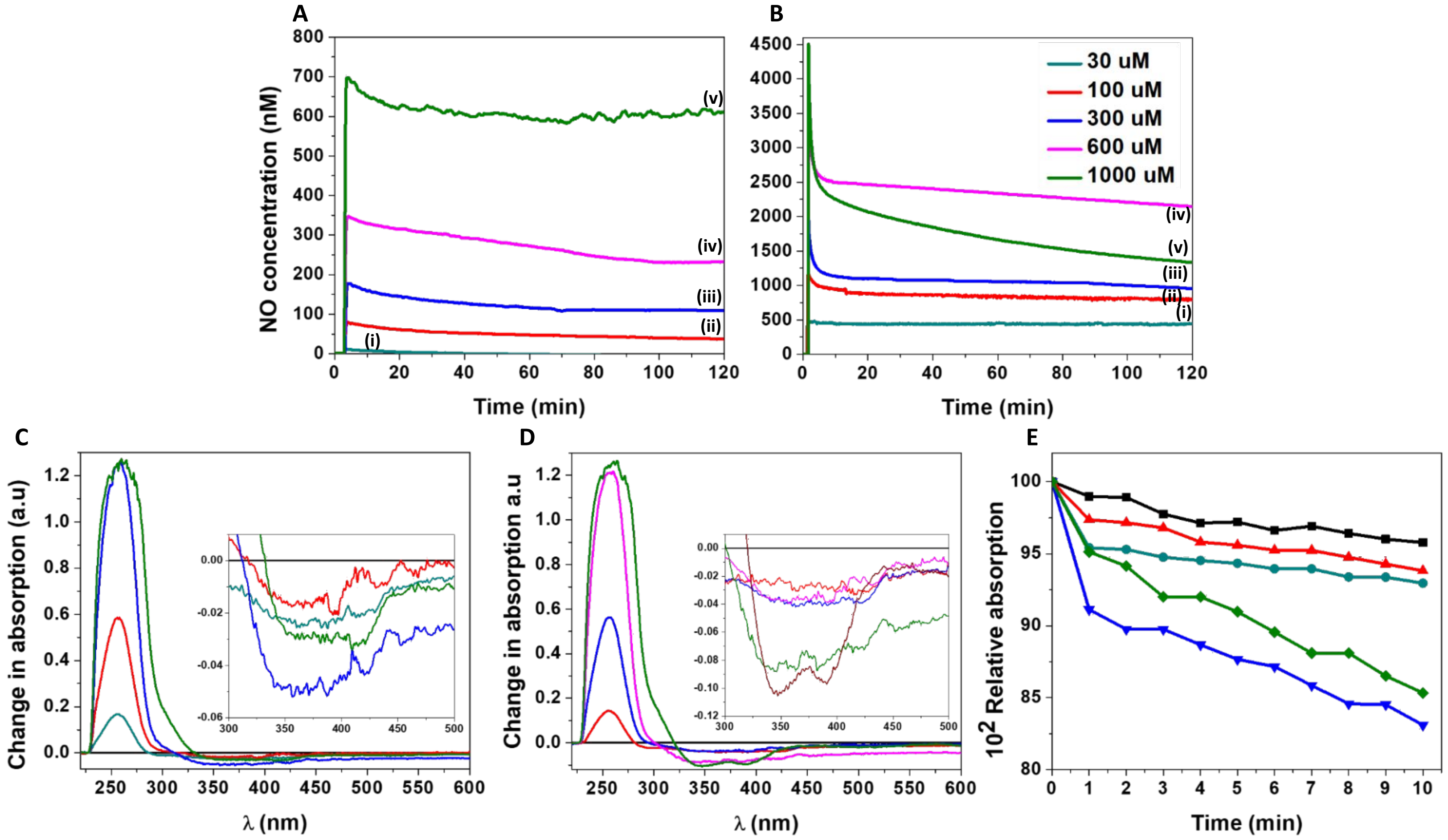

Supplementary Figure S2. •NO flux depends on DETA-NO concentration and decreases the intensity of the Soret band of hemin following binding. (A,B) The average release profile of •NO from 30 (dark cyan, i), 100 (red colour, ii), 300 (blue colour, iii), 600 (magenta colour, iii), and 1000 μM (olive colour, v) DETA-NO in FBS-containing medium and phosphate buffer (50 mM, pH 7.4), respectively. **(C,D)** The difference UV-vis spectra after hemin dilution in the buffer and incubation alone (black colour) or with 30 (dark cyan colour), 100 (red colour), 300 (blue colour) or 1000 μM DETA-NO (olive colour) for one and ten minutes, respectively. The insets: the corresponding spectra within the range 300-500 nm. **(E)** The calculated change in hemin absorption at 382 nm over 10 minutes of incubation. Results are presented as mean values, n = 3.

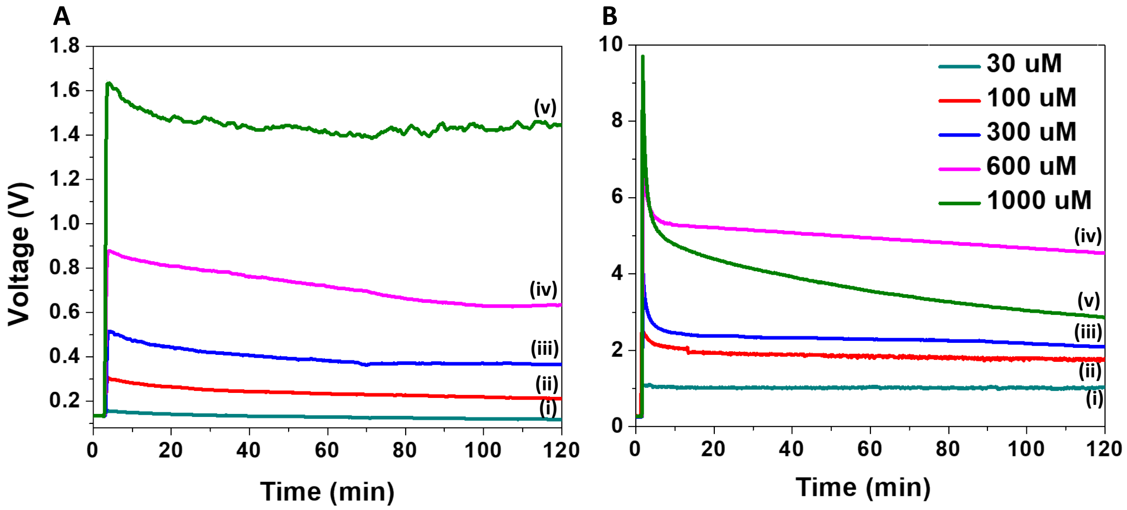

Supplementary Figure S3. Temporal changes in the voltage signal recorded over 120 min following the injection of 30 (dark cyan, i), 100 (red colour, ii), 300 (blue colour, iii), 600 (magenta colour, iii), and 1000 μM (olive colour, v) DETA-NO only into FBS-containing medium **(A)** and phosphate buffer (50 mM, pH 7.4) **(B).** Results are presented as mean voltage readings, n = 3.

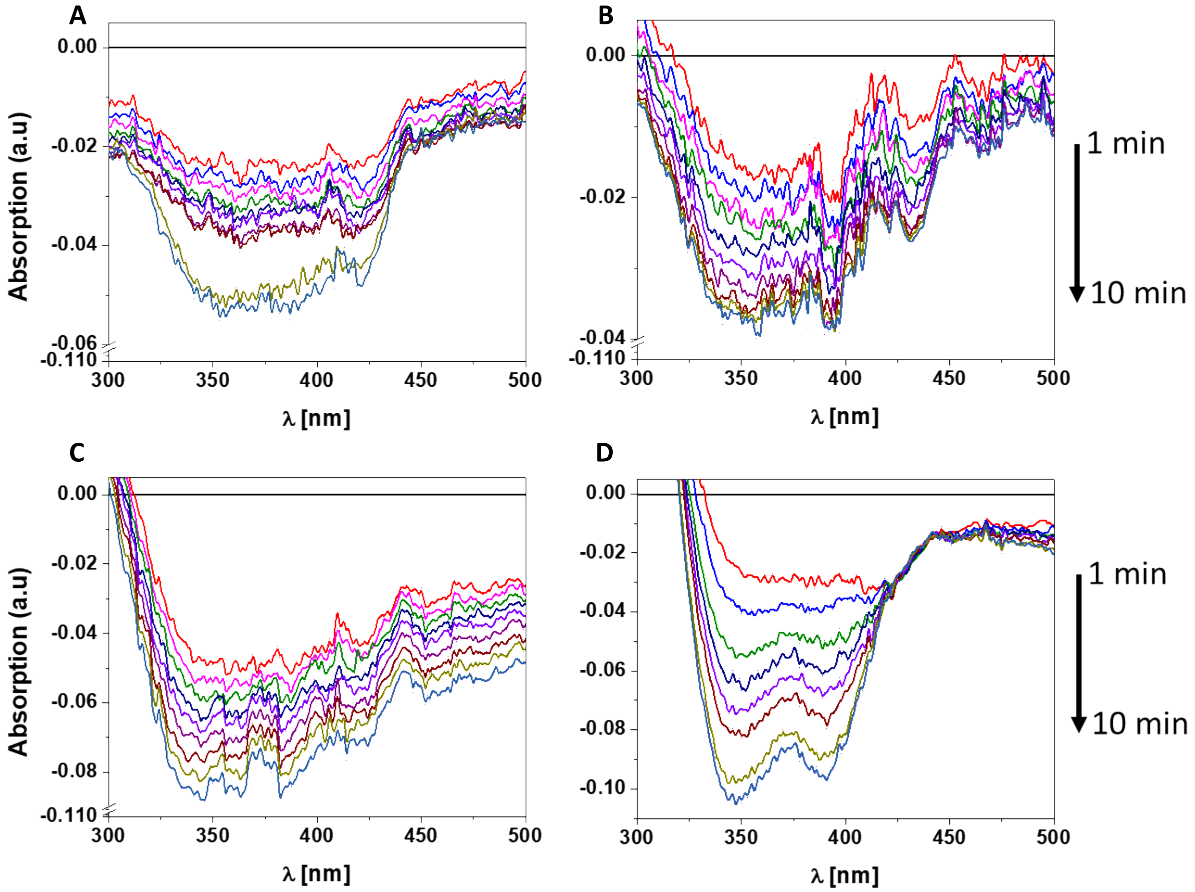

Supplementary Figure S4. Difference UV-vis spectrum of hemin following titration against 30 (**A**), 100 (**B**), 300 (**C**), and 1000 μM (**D**) of DETA-NO in phosphate buffer (50 mM, pH 7.4) over ten minutes. Results are presented as mean values, n = 3.

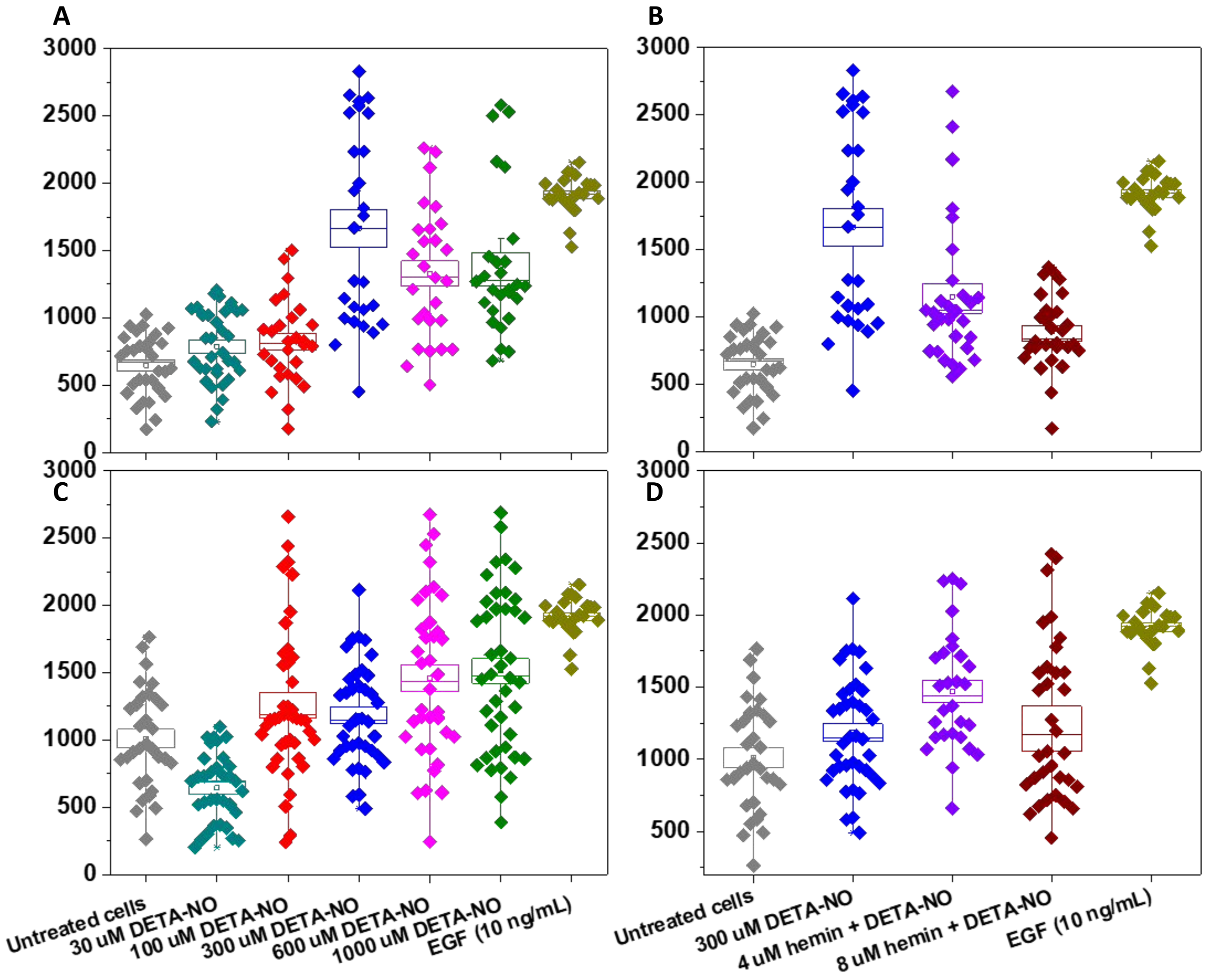

Supplementary Figure S5. Box-whisker blots showing the distribution of the number of counted cells migrated through the transwell membranes towards the chemoattractant composed of FBS-containing RPMI. (**A**,**C**) Effects of different concentrations of DETA-NO and EGF on cell migration (**A**) and invasion through collagen layer (**C**). (**B**,**D**) Effects of DETA-NO with/without hemin and EGF on cell migration (**B**) and invasion through collagen layer (**D**). Results are presented as mean ± S.D, n = 3.

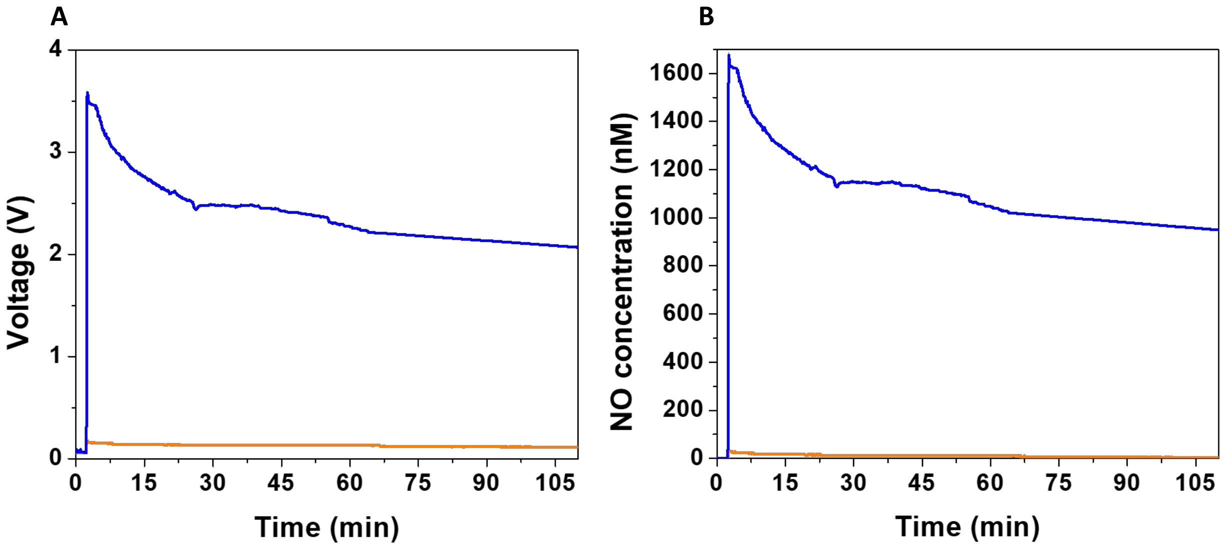

Supplementary Figure S6. Temporal changes in the voltage signal (A) and NO concentration (B) recorded over 110 min following the injection of fresh (blue colour) and degraded DETA-NO (orange colour) into phosphate buffer (50 mM, pH 7.4) with a final concentration of 300 μM. Results are presented as mean values, n = 3.

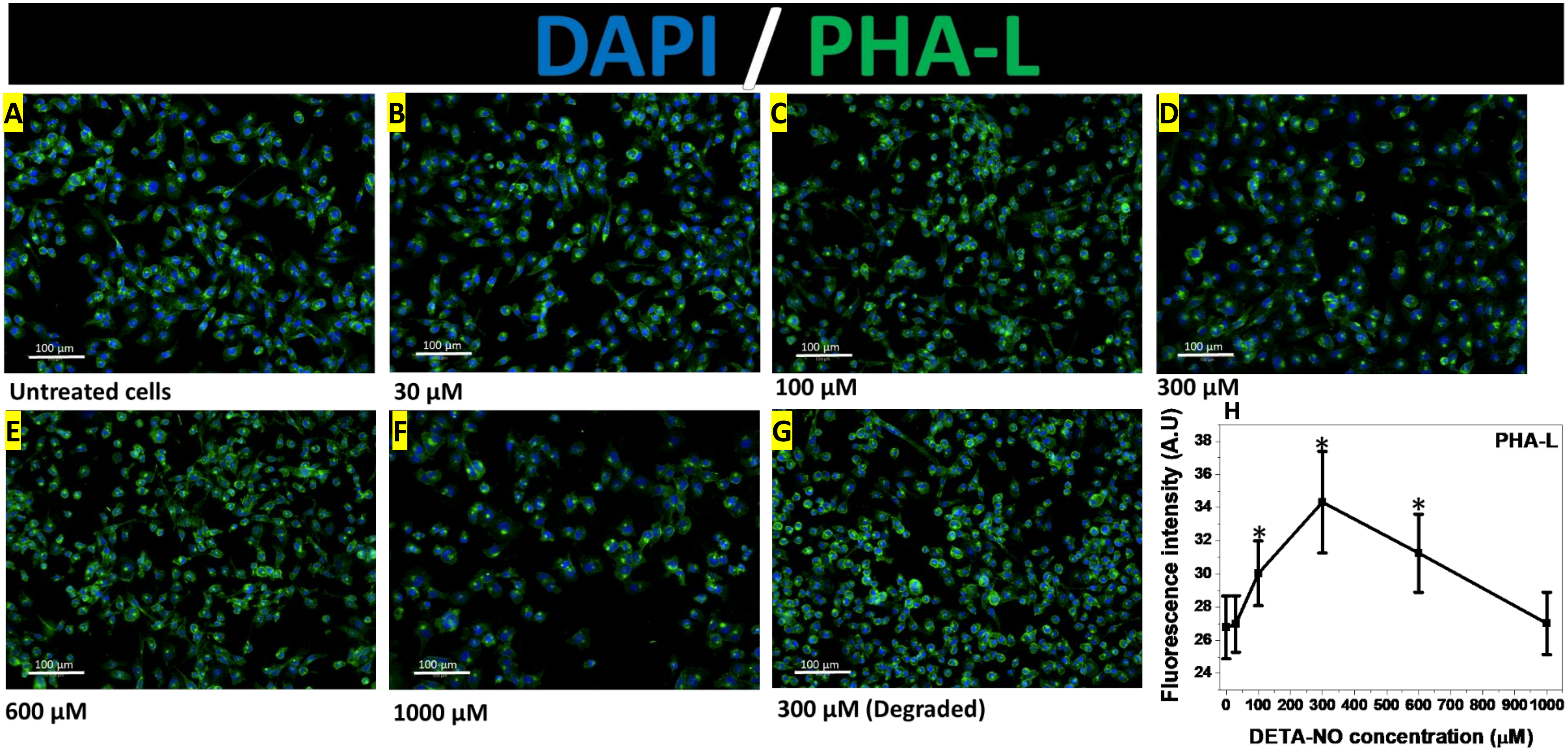

Supplementary Figure S7. Effects of DETA-NO on the expression of PHA-L-binding proteins. MDA-MB-231 cells were treated with medium only **(A)**, fresh 30 **(B)**, 100 **(C)**, 300 **(D)**, 600 **(E)**, 1000 **(F)** or degraded 300 µM DETA-NO **(G)** for 24 hours, then stained with 20 µg/mL FITC-labelled PHA-L lectin. Signals are shown from merge images of the lectin and DAPI staining of nuclei. The fluorescence intensity in 15 areas per well was quantified, normalized to the cell count and averaged for three samples per group **(H).** Results are presented as mean ± S.D. *$P < 0.05$ versus the medium-only treated cells (negative control) using a two-tailed unpaired Student t-test. Scale bar: 100 µm.

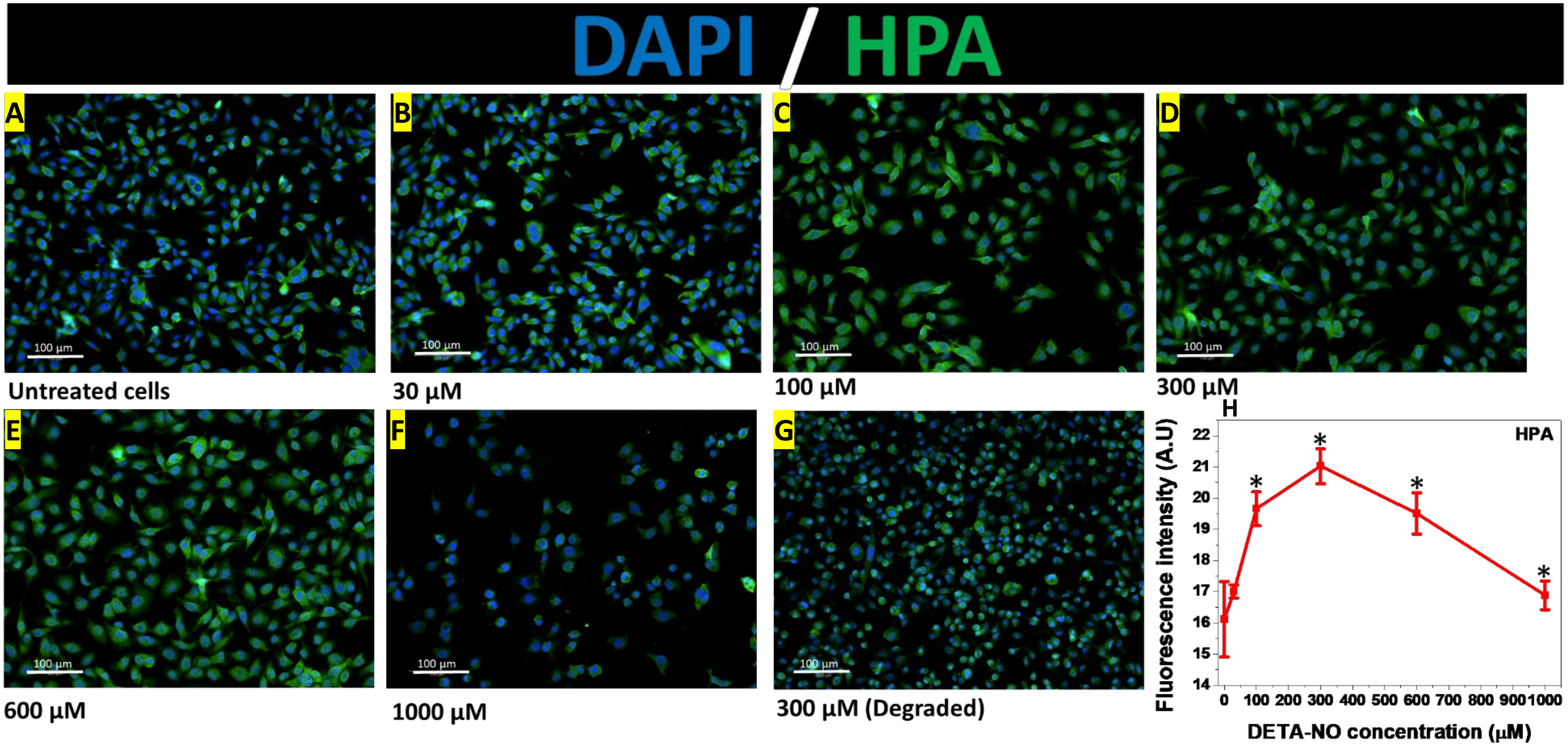

Supplementary Figure S8. Effects of DETA-NO on the expression of HPA-binding proteins. MDA-MB-231 cells were treated with medium only (**A**), fresh 30 (**B**), 100 (**C**), 300 (**D**), 600 (**E**), 1000 (**F**) or degraded 300 µM DETA-NO (**G**) for 24 hours, then stained with 20 µg/mL FITC-labelled HPA lectin. Signals are shown from merge images of the lectin and DAPI staining of nuclei. The fluorescence intensity in 15 areas per well was quantified, normalized to the cell count and averaged for three samples per group (**H**). Results are presented as mean ± S.D. *P < 0.05 versus the medium-only treated cells (negative control) using a two-tailed unpaired Student t-test. Scale bar: 100 µm.

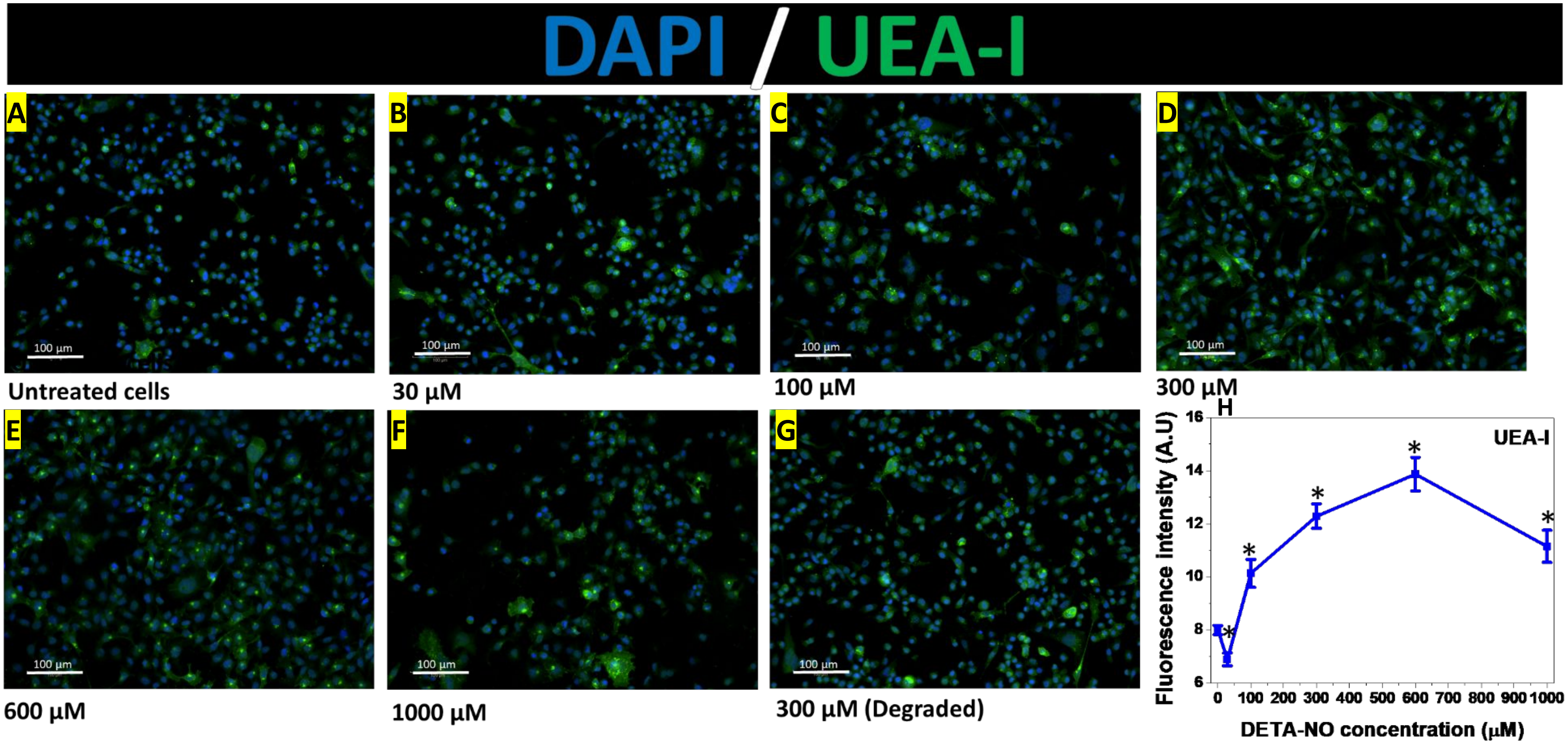

Supplementary Figure S9. Effects of DETA-NO on the expression of UEA-I-binding proteins. MDA-MB-231 cells were treated with medium only (**A**), fresh 30 (**B**), 100 (**C**), 300 (**D**), 600 (**E**), 1000 (**F**) or degraded 300 μM DETA-NO (**G**) for 24 hours, then stained with 20 μg/mL FITC-labelled UEA-I lectin. Signals are shown from merge images of the lectin and DAPI staining of nuclei. The fluorescence intensity in 15 areas per well was quantified, normalized to the cell count and averaged for three samples per group (**H**). Results are presented as mean ± S.D. *$P < 0.05$ versus the medium-only treated cells (negative control) using a two-tailed unpaired Student t-test. Scale bar: 100 μm.

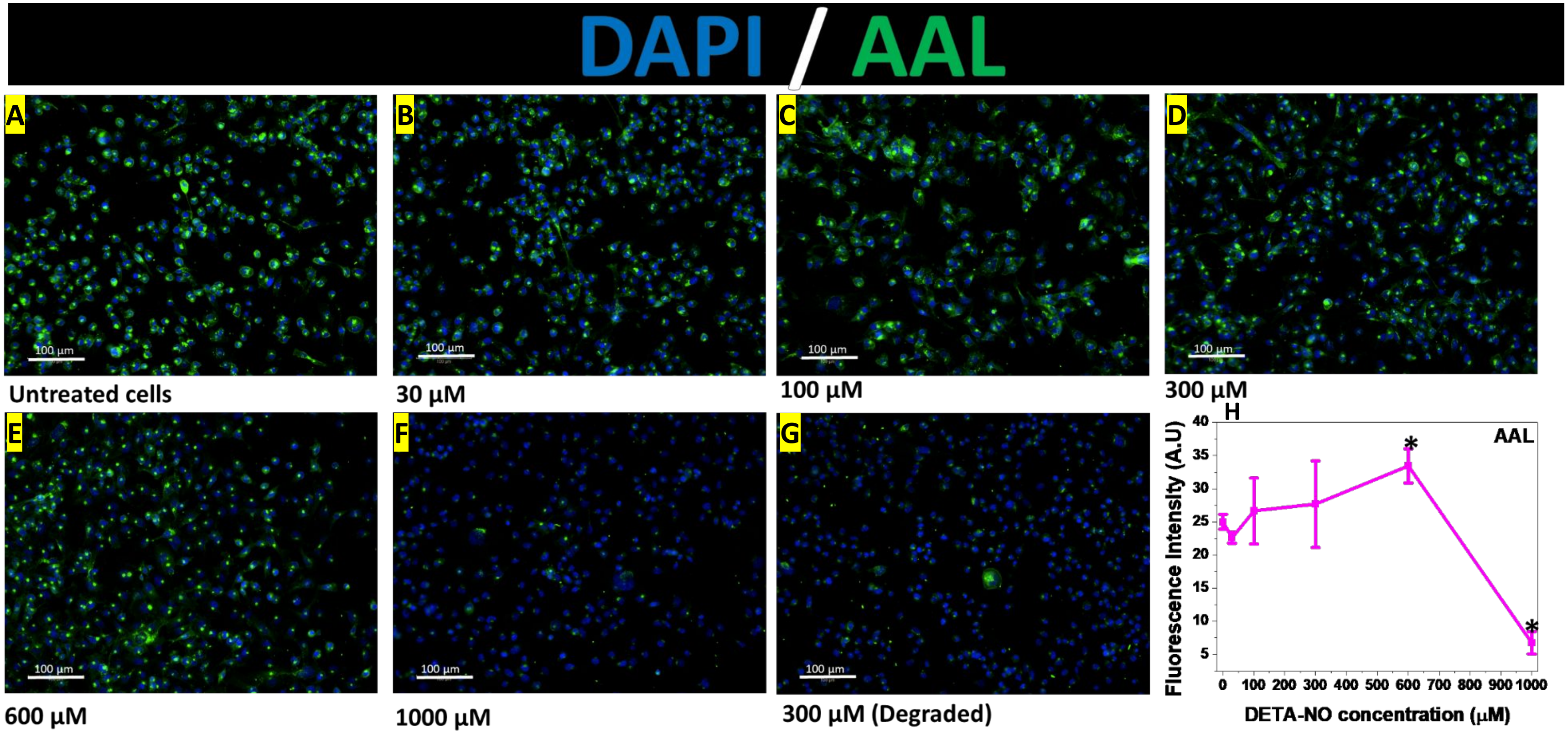

Supplementary Figure S10. Effects of DETA-NO on the expression of AAL-binding proteins. MDA-MB-231 cells were treated with medium only (**A**), fresh 30 (**B**), 100 (**C**), 300 (**D**), 600 (**E**), 1000 (**F**) or degraded 300 µM DETA-NO (**G**) for 24 hours, then stained with 20 µg/mL FITC-labelled AAL lectin. Signals are shown from merge images of the lectin and DAPI staining of nuclei. The fluorescence intensity in 15 areas per well was quantified, normalized to the cell count and averaged for three samples per group (**H**). Results are presented as mean ± S.D. *$P < 0.05$ versus the medium-only treated cells (negative control) using a two-tailed unpaired Student t-test. Scale bar: 100 µm.

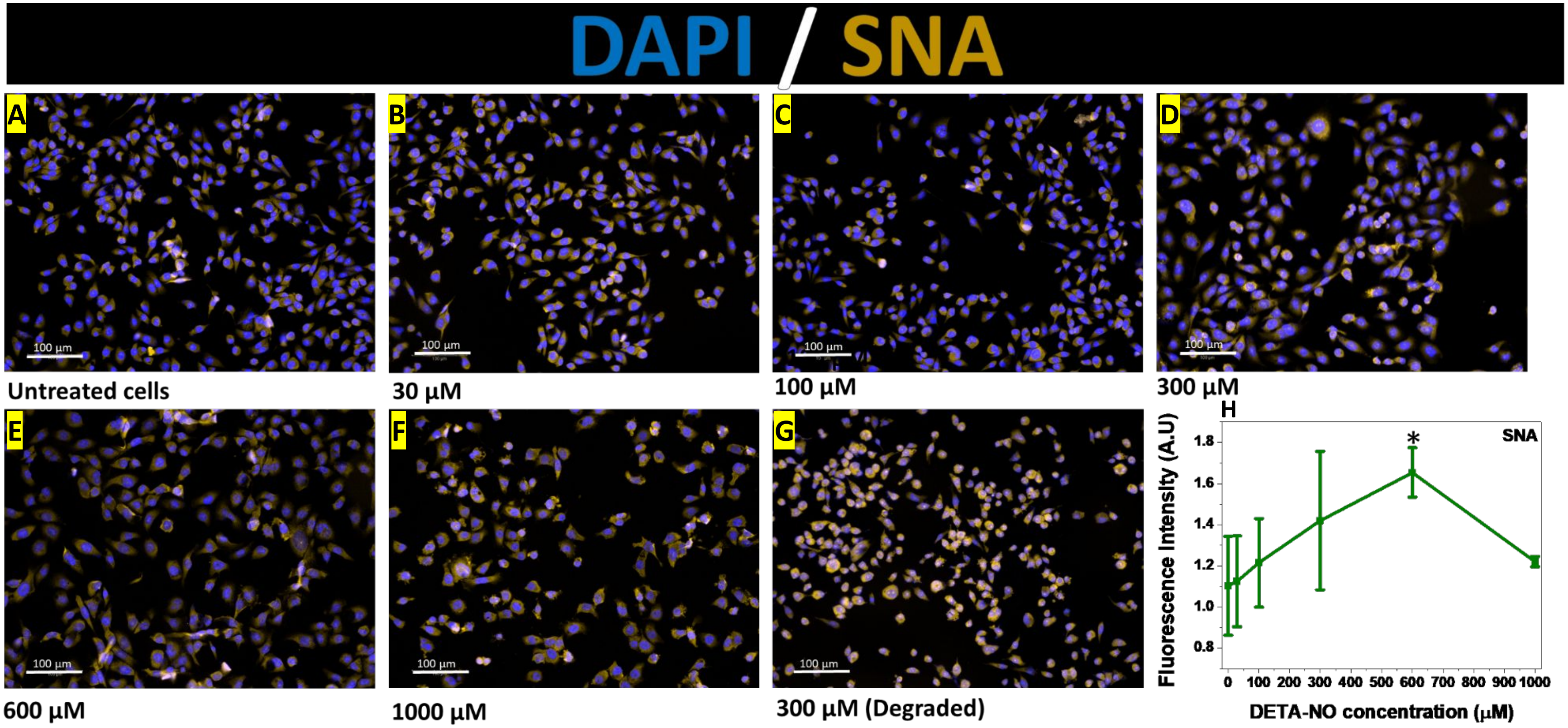

Supplementary Figure S11. Effects of DETA-NO on the expression of SNA-binding proteins. MDA-MB-231 cells were treated with medium only **(A)**, fresh 30 **(B)**, 100 **(C)**, 300 **(D)**, 600 **(E)**, 1000 **(F)** or degraded 300 µM DETA-NO **(G)** for 24 hours, then stained with 20 µg/mL TRITC-labelled SNA lectin. Signals are shown from merge images of the lectin and DAPI staining of nuclei. The fluorescence intensity in 15 areas per well was quantified, normalized to the cell count and averaged for three samples per group **(H).** Results are presented as mean ± S.D. *$P < 0.05$ versus the medium-only treated cells (negative control) using a two-tailed unpaired Student t-test. Scale bar: 100 µm.

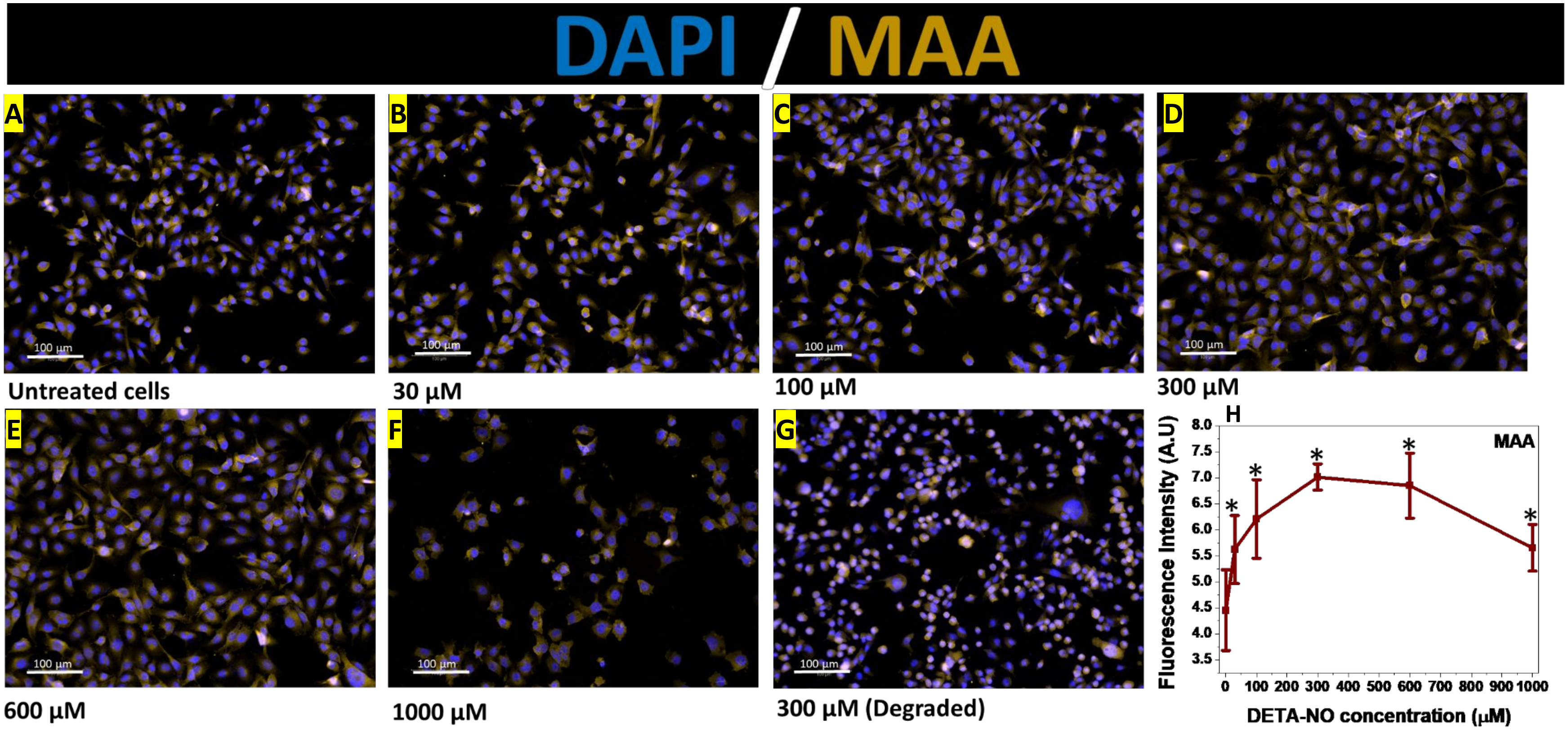

Supplementary Figure S12. Effects of DETA-NO on the expression of SNA-binding proteins. MDA-MB-231 cells were treated with medium only **(A)**, fresh 30 **(B)**, 100 **(C)**, 300 **(D)**, 600 **(E)**, 1000 **(F)** or degraded 300 μM DETA-NO **(G)** for 24 hours, then stained with 20 μg/mL TRITC-labelled SNA lectin. Signals are shown from merge images of the lectin and DAPI staining of nuclei. The fluorescence intensity in 15 areas per well was quantified, normalized to the cell count and averaged for three samples per group **(H).** Results are presented as mean ± S.D. *P < 0.05 versus the medium-only treated cells (negative control) using a two-tailed unpaired Student t-test. Scale bar: 100 μm.

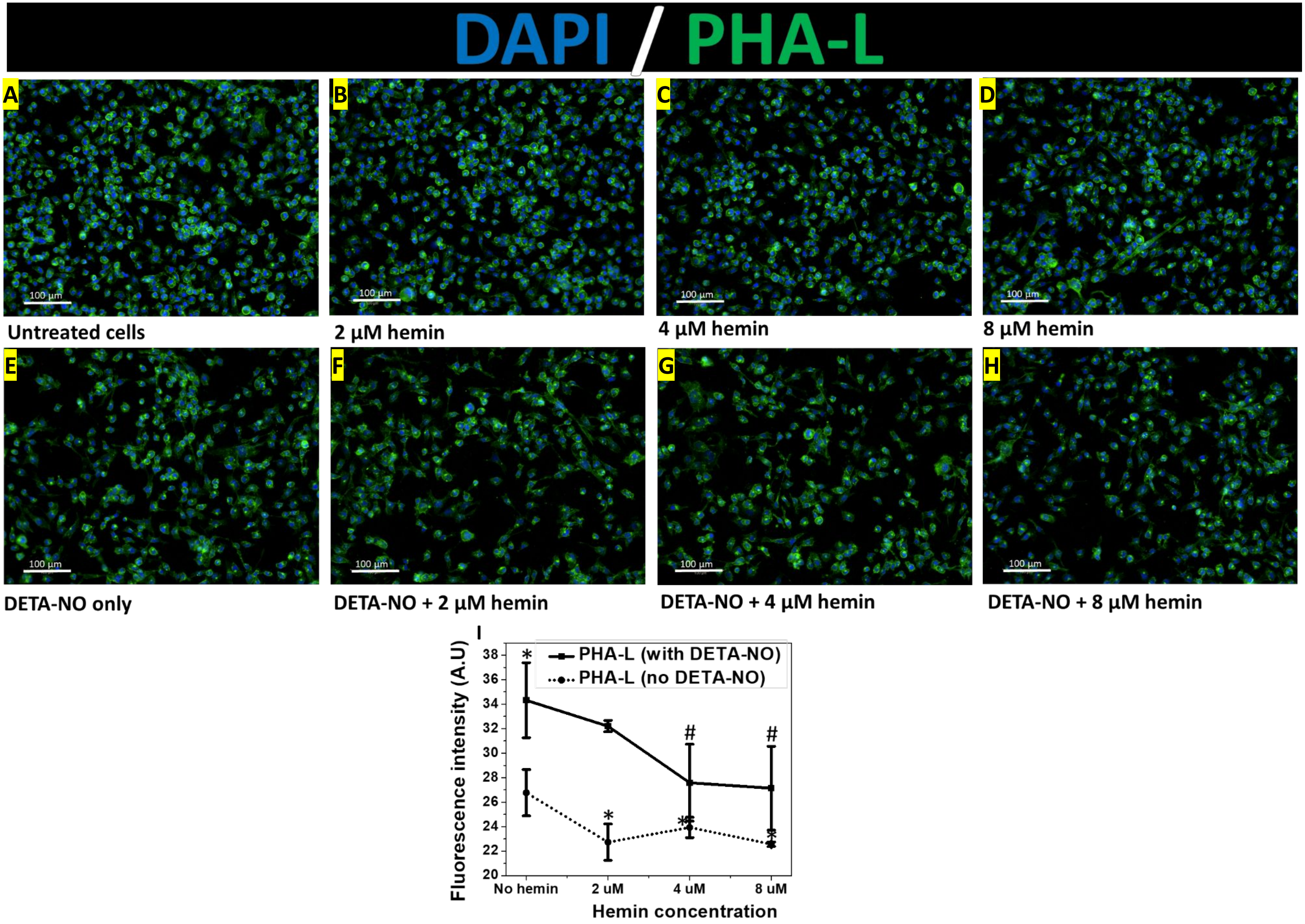
DAPI / PHA-L
A
Untreated cells
B
2 μM hemin
C
4 μM hemin
D
8 μM hemin
E
DETA-NO only
F
DETA-NO + 2 μM hemin
G
DETA-NO + 4 μM hemin
H
DETA-NO + 8 μM hemin
100 μm
I
PHA-L (with DETA-NO)
PHA-L (no DETA-NO)
Fluorescence intensity (A.U)
38
36
34
32
30
28
26
24
22
20
No hemin
2 uM
4 uM
8 uM
Hemin concentration
*
#

Supplementary Figure S13. Effects of hemin and DETA-NO on the expression of PHA-L-binding proteins. MDA-MB-231 cells were treated with medium only (**A**), 2 μM (**B**), 4 μM (**C**), 8 μM hemin (**D**), or 300 μM DETA-NO only (**E**), or a combination of DETA-NO and 2 μM (**F**), 4 μM (**G**), 8 μM hemin (**H**) for 24 hours, then stained with 20 μg/mL FITC-labelled PHA-L lectin. Signals are shown from merge images of the lectin and DAPI staining of nuclei. The fluorescence intensity in 15 areas per well was quantified, normalized to the cell count and averaged for three samples per group **(I).** Results are presented as mean ± S.D. $^{*,\#}$, $P < 0.05$ versus the untreated and DETA-NO only treated cells using a two-tailed unpaired student t-test. Scale bar: 100 μm.

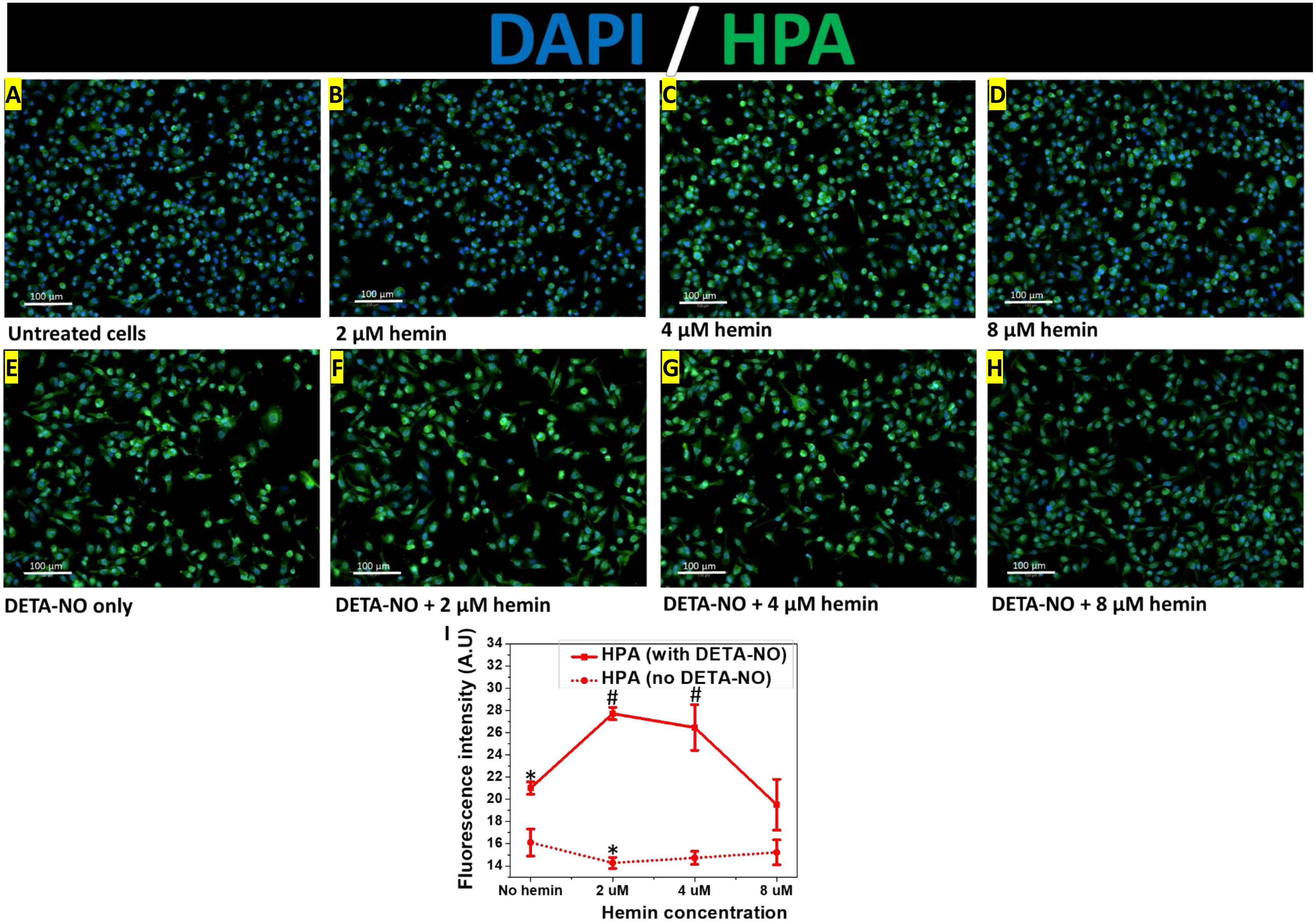
DAPI / HPA
A
B
C
D
100 µm
Untreated cells
2 µM hemin
4 µM hemin
8 µM hemin
E
F
G
H
DETA-NO only
DETA-NO + 2 µM hemin
DETA-NO + 4 µM hemin
DETA-NO + 8 µM hemin
I
HPA (with DETA-NO)
HPA (no DETA-NO)
Fluorescence intensity (A.U)
34
32
30
28
26
24
22
20
18
16
14
#
*
No hemin
2 uM
4 uM
8 uM
Hemin concentration

Supplementary Figure S14. Effects of hemin and DETA-NO on the expression of HPA-binding proteins. MDA-MB-231 cells were treated with medium only (**A**), 2 μM (**B**), 4 μM (**C**), 8 μM hemin (**D**), or 300 μM DETA-NO only (**E**), or a combination of DETA-NO and 2 μM (**F**), 4 μM (**G**), 8 μM hemin (**H**) for 24 hours, then stained with 20 μg/mL FITC-labelled HPA lectin. Signals are shown from merge images of the lectin and DAPI staining of nuclei. The fluorescence intensity in 15 areas per well was quantified, normalized to the cell count and averaged for three samples per group (**I**). Results are presented as mean ± S.D. $^{*,\#}$, $P < 0.05$ versus the untreated and DETA-NO only treated cells using a two-tailed unpaired student t-test. Scale bar: 100 μm.

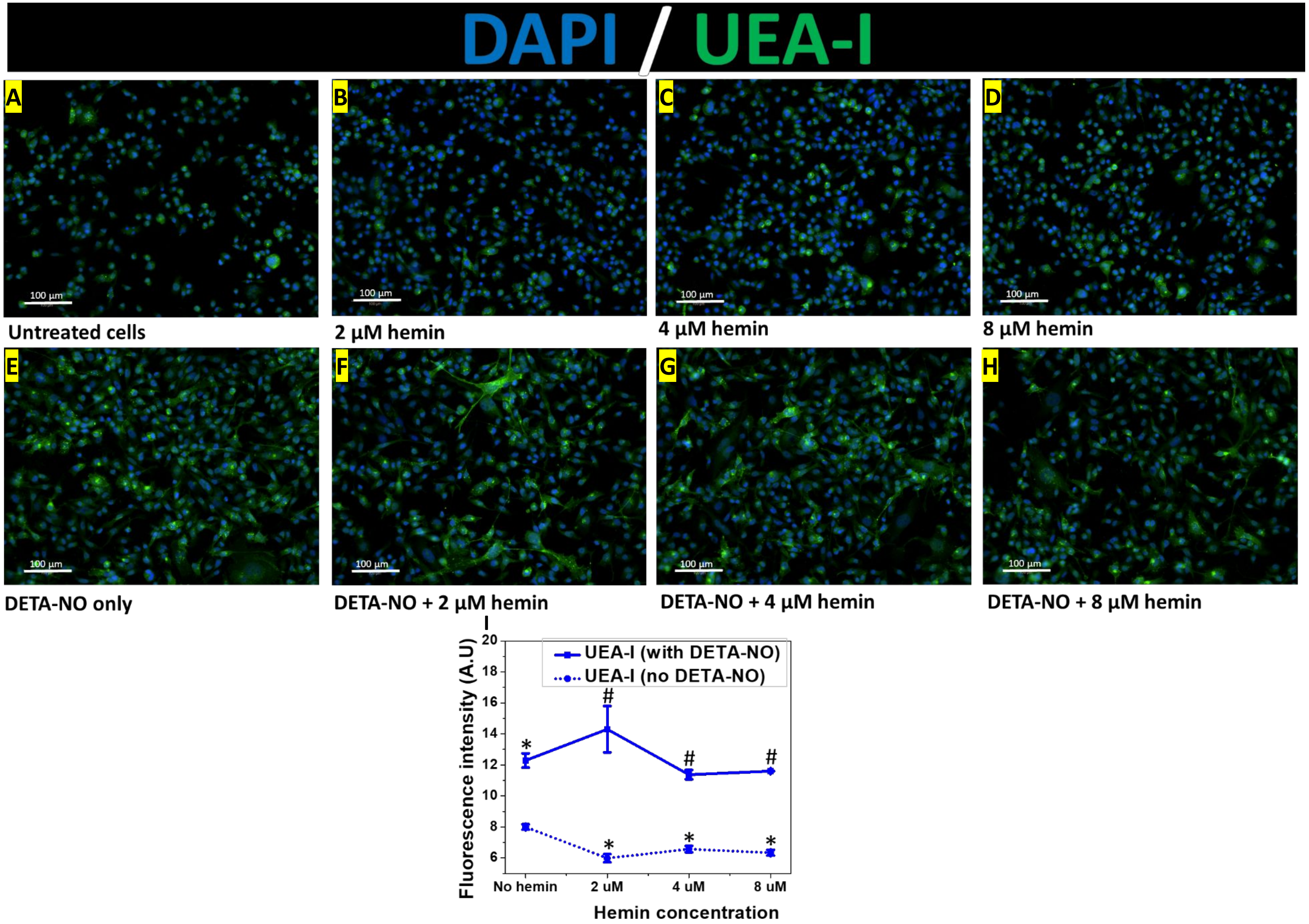
DAPI / UEA-I
A
Untreated cells
B
2 µM hemin
C
4 µM hemin
D
8 µM hemin
E
DETA-NO only
F
DETA-NO + 2 µM hemin
G
DETA-NO + 4 µM hemin
H
DETA-NO + 8 µM hemin
100 µm
I
UEA-I (with DETA-NO)
UEA-I (no DETA-NO)
Fluorescence intensity (A.U)
Hemin concentration
No hemin
2 uM
4 uM
8 uM
20
18
16
14
12
10
8
6
*
#

Supplementary Figure S15. Effects of hemin and DETA-NO on the expression of UEA-I-binding proteins. MDA-MB-231 cells were treated with medium only (**A**), 2 µM (**B**), 4 µM (**C**), 8 µM hemin (**D**), or 300 µM DETA-NO only (**E**), or a combination of DETA-NO and 2 µM (**F**), 4 µM (**G**), 8 µM hemin (**H**) for 24 hours, then stained with 20 µg/mL FITC-labelled UEA-I lectin. Signals are shown from merge images of the lectin and DAPI staining of nuclei. The fluorescence intensity in 15 areas per well was quantified, normalized to the cell count and averaged for three samples per group **(I).** Results are presented as mean ± S.D. $^{*,\#}$, $P < 0.05$ versus the untreated and DETA-NO only treated cells using a two-tailed unpaired student t-test. Scale bar: 100 µm.

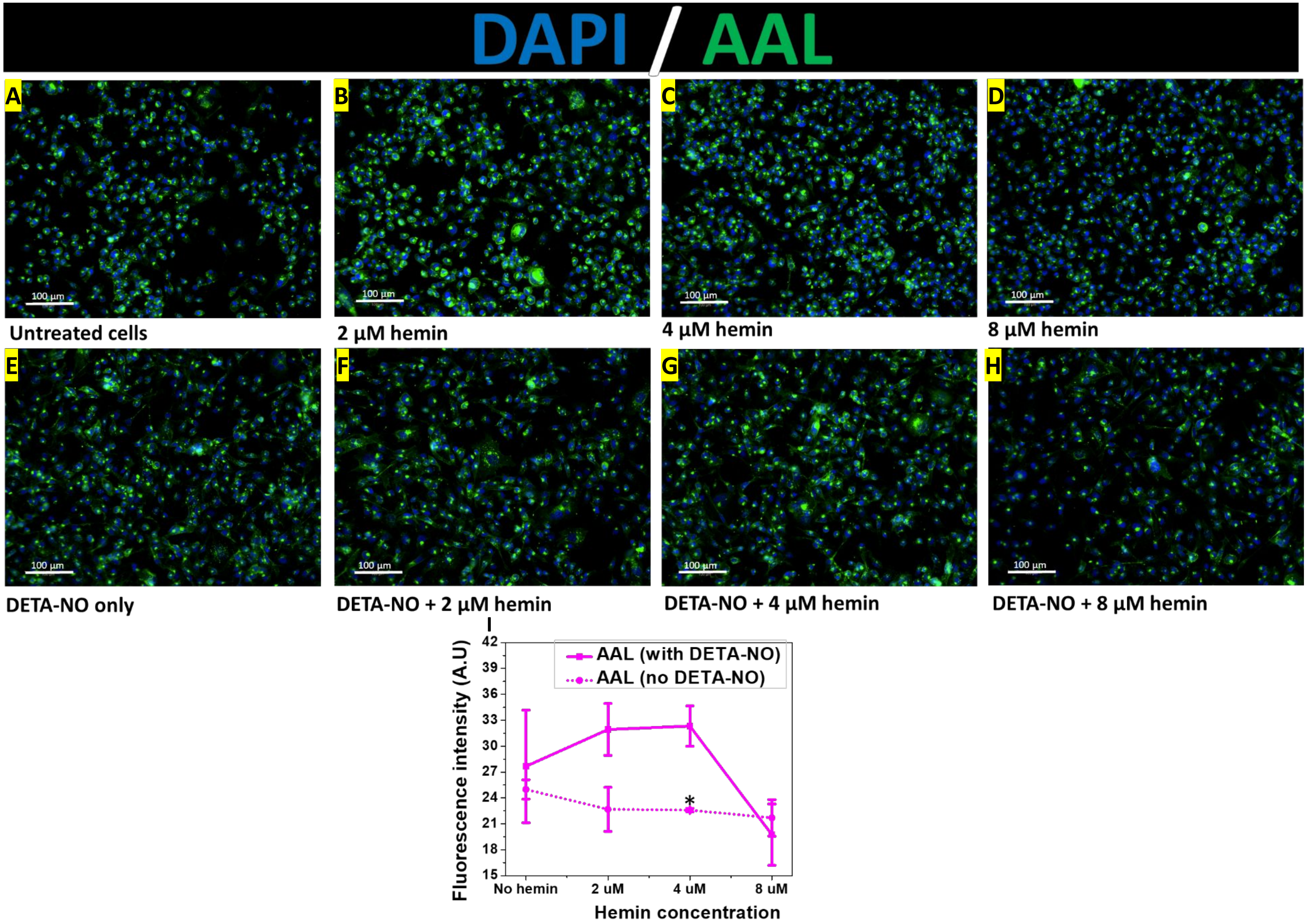
DAPI / AAL
A
100 µm
Untreated cells
B
100 µm
2 µM hemin
C
100 µm
4 µM hemin
D
100 µm
8 µM hemin
E
100 µm
DETA-NO only
F
100 µm
DETA-NO + 2 µM hemin
G
100 µm
DETA-NO + 4 µM hemin
H
100 µm
DETA-NO + 8 µM hemin
I
AAL (with DETA-NO)
AAL (no DETA-NO)
Fluorescence intensity (A.U)
42
39
36
33
30
27
24
21
18
15
*
No hemin
2 uM
4 uM
8 uM
Hemin concentration

Supplementary Figure S16. Effects of hemin and DETA-NO on the expression of AAL-binding proteins. MDA-MB-231 cells were treated with medium only **(A)**, 2 μM **(B)**, 4 μM **(C)**, 8 μM hemin **(D)**, or 300 μM DETA-NO only **(E)**, or a combination of DETA-NO and 2 μM **(F)**, 4 μM **(G)**, 8 μM hemin **(H)** for 24 hours, then stained with 20 μg/mL FITC-labelled AAL lectin. Signals are shown from merge images of the lectin and DAPI staining of nuclei. The fluorescence intensity in 15 areas per well was quantified, normalized to the cell count and averaged for three samples per group **(I).** Results are presented as mean ± S.D. *,#, $P < 0.05$ versus the untreated and DETA-NO only treated cells using a two-tailed unpaired student t-test. Scale bar: 100 μm.

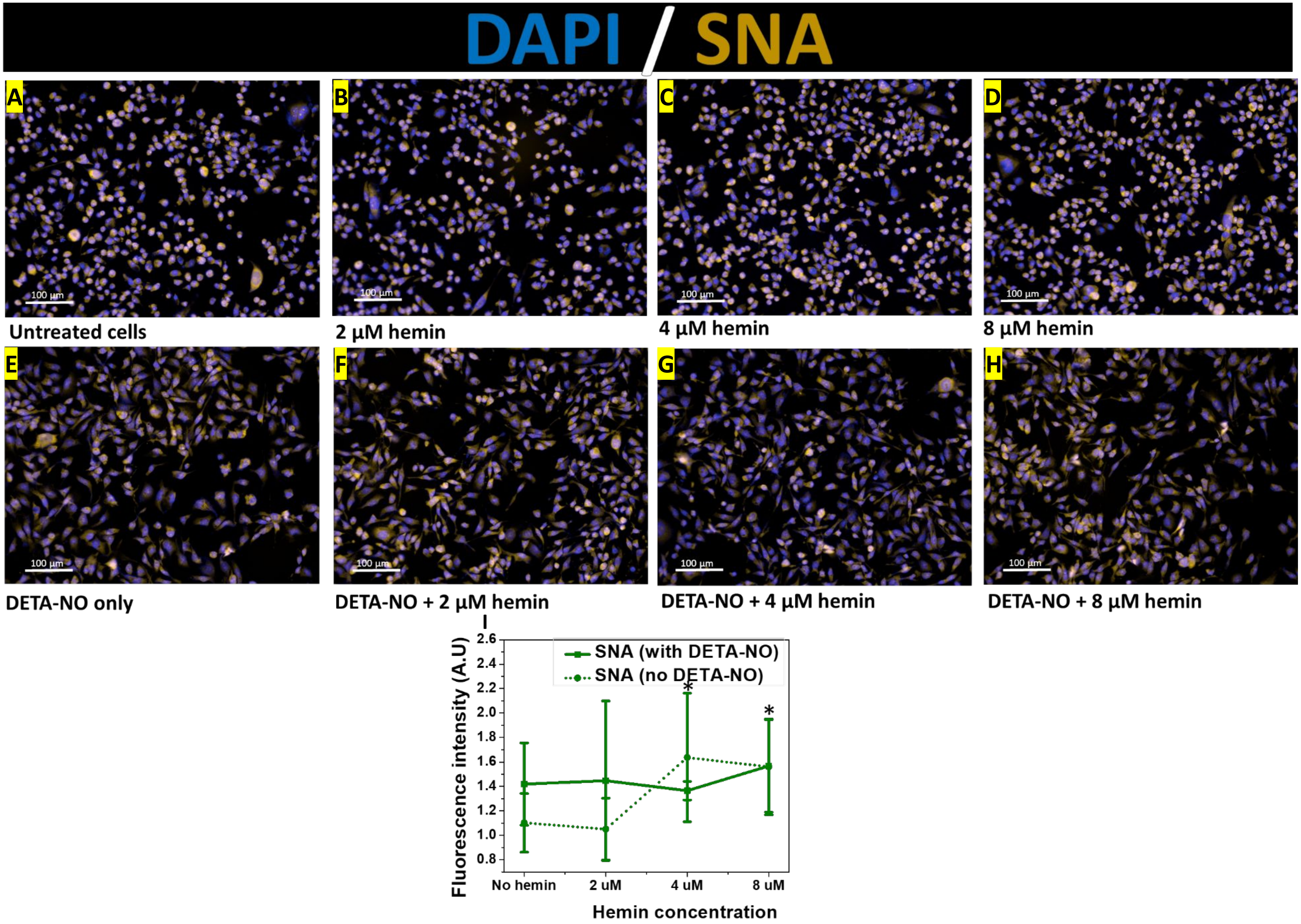
DAPI / SNA
A
B
C
D
100 µm
Untreated cells
2 µM hemin
4 µM hemin
8 µM hemin
E
F
G
H
DETA-NO only
DETA-NO + 2 µM hemin
DETA-NO + 4 µM hemin
DETA-NO + 8 µM hemin
SNA (with DETA-NO)
SNA (no DETA-NO)
Fluorescence intensity (A.U)
2.6
2.4
2.2
2.0
1.8
1.6
1.4
1.2
1.0
0.8
No hemin
2 uM
4 uM
8 uM
Hemin concentration
*
*

Supplementary Figure S17. Effects of hemin and DETA-NO on the expression of SNA-binding proteins. MDA-MB-231 cells were treated with medium only **(A)**, 2 μM **(B)**, 4 μM **(C)**, 8 μM hemin **(D)**, or 300 μM DETA-NO only **(E)**, or a combination of DETA-NO and 2 μM **(F)**, 4 μM **(G)**, 8 μM hemin **(H)** for 24 hours, then stained with 20 μg/mL TRITC-labelled SNA lectin. Signals are shown from merge images of the lectin and DAPI staining of nuclei. The fluorescence intensity in 15 areas per well was quantified, normalized to the cell count and averaged for three samples per group **(I).** Results are presented as mean ± S.D. $^{*,\#}$, $P < 0.05$ versus the untreated and DETA-NO only treated cells using a two-tailed unpaired student t-test. Scale bar: 100 μm.

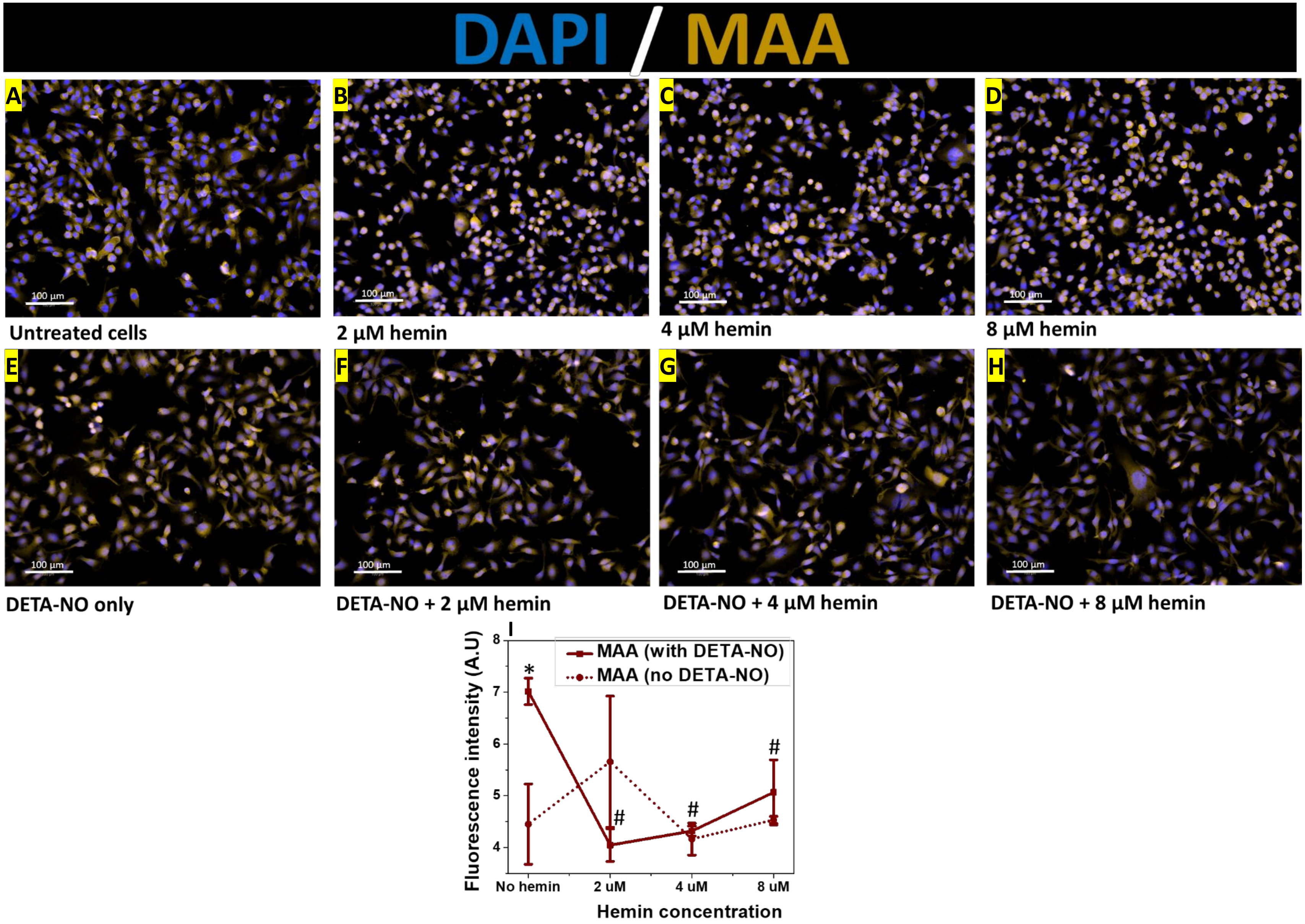
DAPI / MAA
A
B
C
D
100 µm
Untreated cells
2 µM hemin
4 µM hemin
8 µM hemin
E
F
G
H
DETA-NO only
DETA-NO + 2 µM hemin
DETA-NO + 4 µM hemin
DETA-NO + 8 µM hemin
I
MAA (with DETA-NO)
MAA (no DETA-NO)
Fluorescence intensity (A.U)
Hemin concentration
No hemin
2 uM
4 uM
8 uM
*
#

Supplementary Figure S18. Effects of hemin and DETA-NO on the expression of SNA-binding proteins. MDA-MB-231 cells were treated with medium only **(A)**, 2 μM **(B)**, 4 μM **(C)**, 8 μM hemin **(D)**, or 300 μM DETA-NO only **(E)**, or a combination of DETA-NO and 2 μM **(F)**, 4 μM **(G)**, 8 μM hemin **(H)** for 24 hours, then stained with 20 μg/mL TRITC-labelled SNA lectin. Signals are shown from merge images of the lectin and DAPI staining of nuclei. The fluorescence intensity in 15 areas per well was quantified, normalized to the cell count and averaged for three samples per group **(I)**. Results are presented as mean ± S.D. *,#, P < 0.05 versus the untreated and DETA-NO only treated cells using a two-tailed unpaired student t-test. Scale bar: 100 μm.

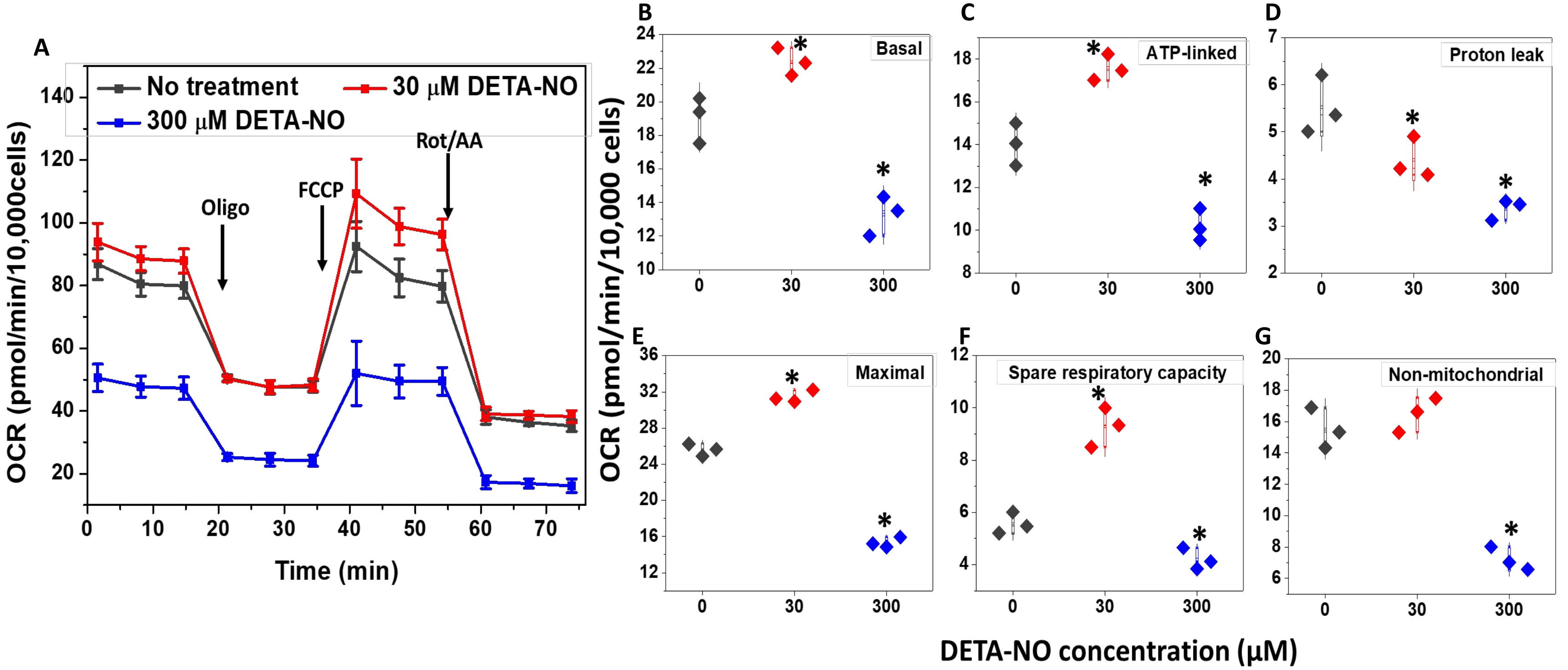

Supplementary Figure S19. The impact of 24-hour treatment with DETA-NO on the mitochondrial function of MDA-MB-231 cell measured by Mito Stress test. **(A)** The representative kinetic plot, shows the changes in OCR values following cell treatment with 30 and 300 μM DETA-NO and incubation for 24 hours, followed by the normal procedures of the assay. The individual parameters of mitochondrial function were summarized as basal **(B)**, ATP-linked **(C)**, proton leak **(D)**, maximal respiration **(E)**, spare respiratory capacity **(F)** and non-mitochondrial-OCR **(G)**. Results are presented as mean ± S.D, n = 3. *$p < 0.05$ compared to the untreated cells using a two-tailed unpaired student t-test. This experiment was repeated two times.

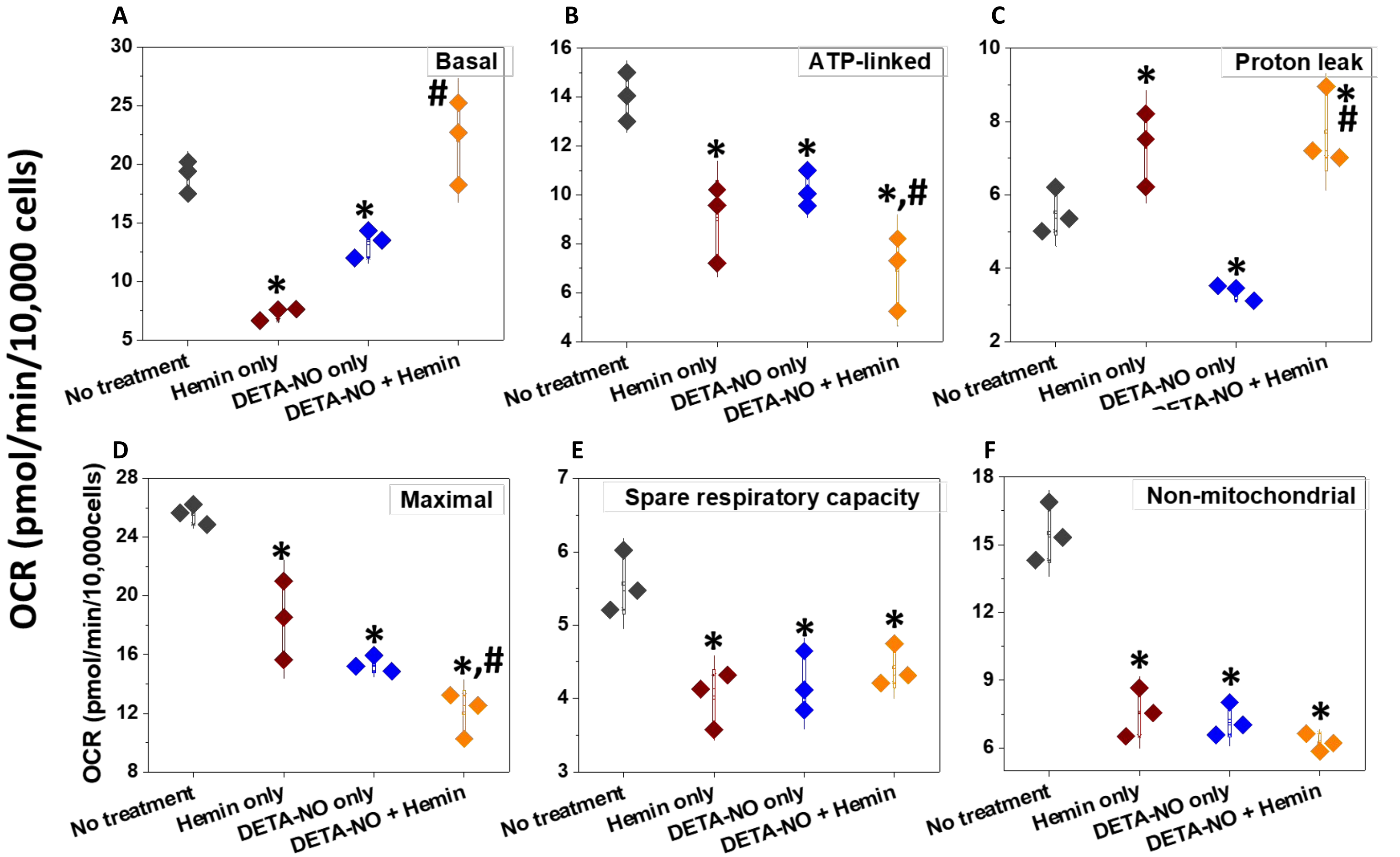

Supplementary Figure S20. The impact of 24-hour treatment with DETA-NO and/or hemin on the mitochondrial function of MDA-MB-231 cell measured by Mito Stress test. The cells were treated with 300 μM DETA-NO and/or 8 μM hemin to cells and incubated for 24 hours, followed by the normal procedures of the assay. The individual parameters of mitochondrial function were summarized as basal (**A**), ATP-linked (**B**), proton leak (**C**), maximal respiration (**D**), spare respiratory capacity (**E**) and non-mitochondrial-OCR (**F**). Results are presented as mean ± S.D, n = 3. *p < 0.05 compared to the untreated cells using a two-tailed unpaired student t-test. This experiment was repeated two times.
